# Stick or Spill? Scaling Relationships for the Binding Energies of Adsorbates on Single-Atom Alloy Catalysts

**DOI:** 10.1021/acs.jpclett.2c01519

**Published:** 2022-08-02

**Authors:** Romain Réocreux, E. Charles H. Sykes, Angelos Michaelides, Michail Stamatakis

**Affiliations:** † Thomas Young Centre and Department of Chemical Engineering, University College London, Roberts Building, Torrington Place, London WC1E 7JE, U.K.; ‡ Department of Chemistry, Tufts University, Medford, Massachusetts 02155, United States; § Yusuf Hamied Department of Chemistry, University of Cambridge, Lensfield Road, Cambridge CB2 1EW , U.K.

## Abstract

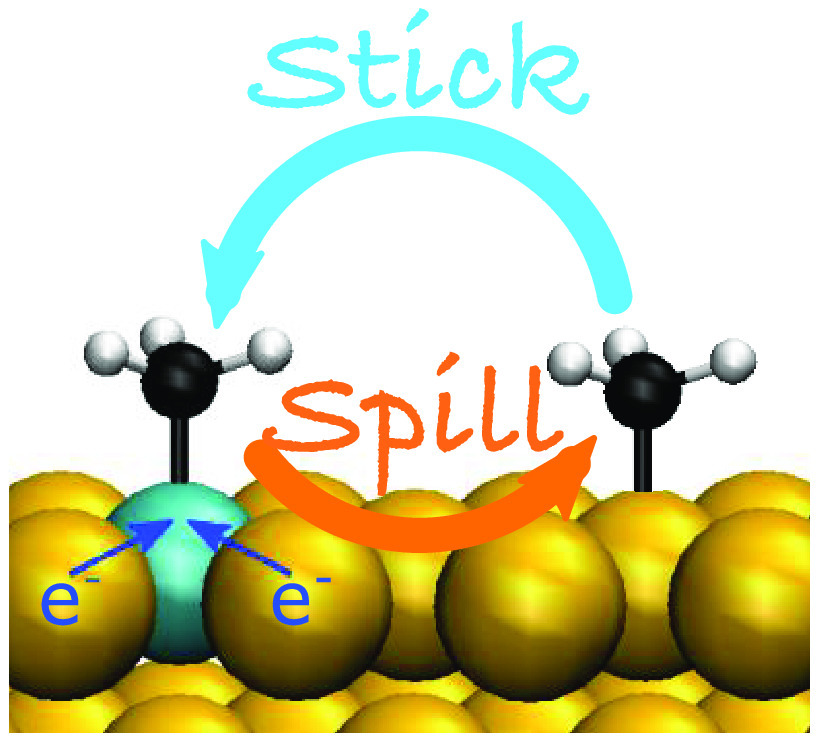

Single-atom alloy catalysts combine catalytically active metal atoms, present as dopants, with the selectivity of coinage metal hosts. Determining whether adsorbates stick at the dopant or spill over onto the host is key to understanding catalytic mechanisms on these materials. Despite a growing body of work, simple descriptors for the prediction of spillover energies (SOEs), i.e., the relative stability of an adsorbate on the dopant versus the host site, are not yet available. Using Density Functional Theory (DFT) calculations on a large set of adsorbates, we identify the dopant charge and the SOE of carbon as suitable descriptors. Combining them into a linear surrogate model, we can reproduce DFT-computed SOEs within 0.06 eV mean absolute error. More importantly, our work provides an intuitive theoretical framework, based on the concepts of electrostatic interactions and covalency, that explains SOE trends and can guide the rational design of future single-atom alloy catalysts.

Single-atom alloys (SAAs), which consist of atomically dispersed metal atoms doped typically in the surface of Cu, Ag, or Au nanoparticles, are emerging as highly active and selective catalysts.^1−5^ Their catalytic performance is closely related to their ability to direct selected adsorbates from host sites to dopant sites, where difficult elementary steps can be catalyzed, and redirect the products to the host sites or the gas phase to prevent any poisoning. Although entropy can play a role in a limited number of cases (see Role of Entropy in the Supporting Information), the driving force for this crucial surface migration is the binding energy difference of adsorbates between host and dopant sites, also referred to as the spillover energy (SOE). For example, surface Pd dopants efficiently split H_2_(ads) to 2H(ads). The SOE of H(ads) is however more favorable on the PdCu SAA than on the PdAu SAA,^6^ resulting in the former being better than the latter as a hydrogenation catalyst.^7^ Spectator species with large SOEs have also been shown to modulate the performance of SAA catalysts, with CO(ads) or I(ads) being prominent examples of these effects,^8,9^ or even to stabilize SAA surfaces by anchoring dopants at the surface of the catalyst.^4,10−13^ Therefore, the ability to understand factors affecting the SOEs and predict SOEs for a wide range of chemical intermediates on various SAA surfaces is key to the development of this new class of catalysts.

The binding energy of a species to a given adsorption site (the SOE is the difference between two binding energies) is routinely computed with Density Functional Theory (DFT) calculations.^4,9^ Machine-learning models, trained on DFT data, have also emerged, and they can accelerate the elucidation of new SAA materials and reactions.^14−18^ Albeit accurate, these models provide extremely complex nonlinear multiparameter relationships that are typically specific to a given problem (e.g., a given elementary step) and do not perform well upon extrapolation beyond the domain spanned by the training data set. The field would therefore benefit from simple, widely applicable, descriptor-based models that capture trends relative to the SOEs on SAAs. This would help narrow down, intuitively, the most promising SAA candidates in terms of the best host metal and dopant atom combinations, considering the stability of the alloy itself and the optimal energetic pathways for the targeted reaction.

In the past, the development of metal catalysts (monometallic and intermetallic) has benefitted greatly from descriptor-based linear scaling relationships including thermochemical scaling relationships,^19^ Brønsted–Evans–Polanyi relationships,^20−22^ the d-band model,^23,24^ and relationships using the generalized coordination number.^25^ The success of these models results from their simplicity: only a few parameters (e.g., the d-band center or the binding energies of C and O) are needed to provide semiquantitative predictions regarding the performance of catalysts.^16,26−28^ These models have proved particularly useful for experimentalists and theoreticians to rationalize experimental observations^24,27^ and predict behaviors *on-the-fly* in multiscale modeling simulations.^29^ Although some of these simple relationships hold for SAAs, the behavior of these materials can significantly differ from that of pure transition metals.^30,31^ This can be attributed to the unique electronic structure of SAAs, which has been compared to that of gas phase atoms.^30^ In this regard, descriptors commonly used in molecular chemistry (*e.g.,* molecular orbitals, atomic charges) have been considered to rationalize the behavior of SAAs.^32−37^ There are examples in the literature where charge analyses on DFT calculations were performed to explain experimental shifts observed in X-ray absorption and photoemission spectra.^38,39^ However, the origin of these charges on the dopant atom and their impact on the stability of chemical intermediates adsorbed on the dopant site of SAAs (electrostatic repulsion/attraction) are yet to be understood.

Here, we consider the SAA dopant atomic charge as a potential descriptor for the SOE of a broad range of catalytically relevant adsorbates (H, CO, OH_*x*_, NH_*y*_, and CH_*z*_). We have performed plane-wave DFT calculations, using the optB86b-vdW functional^40^ on periodic models of the most stable (111) facet of 12 SAAs (Figure 1a). We have computed the atomic charges of Cu-, Ag- and Au-based SAAs doped with Ni, Pd, Rh, and Pt, following the approach developed by Bader (details in the Supporting Information).^41^ As shown in parts b and c of Figure 1, the dopant atom can exhibit a significant atomic charge, ranging from −0.61*e* for PtCu to +0.32*e* for NiAu (*e*, taken as positive, being the elementary charge). The countercharge is delocalized over the host atoms of the slab making their atomic charges negligible (Table S1). As a general trend, the dopant sites of Cu-based SAAs are more negatively charged than Ag- and even more so than Au-based SAAs (Figure 1b,c). Figure 1d shows that the atomic charges indeed correlate linearly with Pauling’s electronegativity difference (data in Table S2). The more electronegative element retrieves a fraction of an electron from the less electronegative one. Copper, as the least electronegative host (χ_*Cu*_ = 1.90), favors anionic dopants, whereas gold, the most electronegative element of the d-block (χ_*Au*_ = 2.54), promotes more cationic dopants. This trend holds, to some extent, when considering other partitioning schemes for the definition of atomic charges. The refined Density Derived Electrostatic and Chemical (DDEC6) approach^42,43^ and the Hirshfeld-Dominant (HD) method^44^ both confirm that Cu and Ag hosts donate more electron density to the dopant atoms than Au hosts (Table S1).

**Figure 1:**
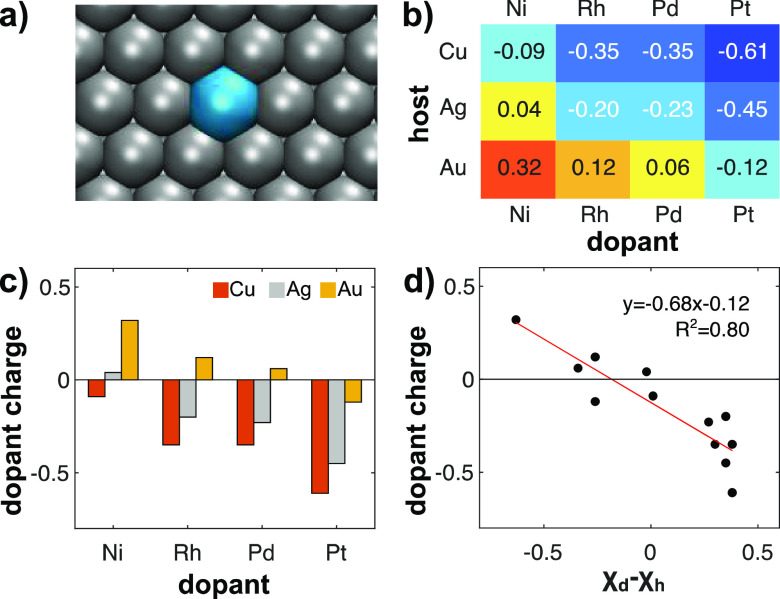
Atomic charges (Bader analysis) in SAA surfaces. (a) Surface structure of the (111) facet of a SAA (dopant in cyan, host in gray). (b) Heatmap chart and (c) plot of the dopant charges (in units of *e*, the elementary charge) for Cu-, Ag-, and Au-based SAAs. (d) Correlation between the dopant charge and the electronegativity (χ) difference between the host (χ_*h*_) and the dopant (χ_*d*_) metals.

Using Bader analysis, we have considered the impact of the dopant charge on the relative stability of adsorbates between guest and host sites, the host sites being essentially uncharged due to delocalization. To this end, we have computed the SOE for a variety of catalytically relevant adsorbates (Tables S3 and S4 and Figures 2 and S2). The SOE is defined as the energy required for an adsorbate to migrate from the dopant atom site to the most stable site on the host metal (Figure 2a). The SOE can therefore be calculated from the formation energies of the adsorbate on host sites *E*_*f*,*h*_ and dopant sites *E*_*f*,*d*_ (Table S3). It can also be calculated from the adsorption energies on host sites Δ_*ads*_*E*_*h*_ and dopant sites Δ_*ads*_*E*_*d*_ (eq i).
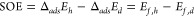
iFocusing first on OH_*x*_, CH_3_OH, and NH_*y*_ (Figure 2c,d), we can see that there is a certain degree of correlation between the SOE and the SAA dopant charge. Even if the quality of the correlation, which will be discussed later, is not equal for all adsorbates, the SOE generally increases with the dopant charge. Interestingly, all these adsorbates bind to the surface via their heteroatom (N or O) that carries a negative partial charge. Thus, it is expected, from an electrostatic perspective, that more negatively charged dopants would bind these adsorbates less strongly than the essentially uncharged host, thereby decreasing the SOE. One striking example of this effect is that OH has negative SOEs (higher stability on host sites) for all SAAs with a dopant charge <−0.21*e*. Other similar examples can be found in Table S4, and together, these data indicate that the dopant charge should be taken into consideration when developing SAA catalysts. The correlation between the SOE and the dopant charge holds for saturated (CH_3_OH, H_2_O, NH_3_) or nearly saturated (OH) adsorbates (*R*^2^ ≥ 0.65) and deteriorates for highly unsaturated species (*e.g.,* N and C). To get insight into the nature of the binding mechanism of the different adsorbates on SAAs, one can tentatively decompose the SOE into three terms: a dispersion term Δ*E*_*vdw*_, an electrostatic term Δ*E*_*elec*_, and a covalent term Δ*E*_*cov*_ (eq ii). Comparing the SOEs of OH computed with two well-established functionals, namely PBE and optB86b-vdW,^40,45^ is insightful. The correlation part of the latter functional is specifically designed to account for dispersion interactions, which are poorly described by the former. Despite this essential difference, the two sets of SOEs for OH are similar within 0.04 eV. This suggests that the dispersion contribution Δ*E*_*vdw*_ can be neglected in the energetic decomposition (Table S4 and eq iii).
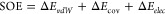
ii
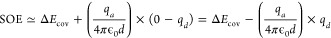
iiiThe electrostatic contribution on host sites in assumed to be zero as the host is not charged. In eq iii, ϵ_0_ is the vacuum permittivity, *q*_*a*_ the atomic charge of the adsorbate’s heteroatom, *q*_*d*_ the atomic charge of the dopant atom, and *d* the distance between the adsorbate and the dopant. Assuming that the interaction is essentially ionic, i.e., electrons are localized on each species and not shared via a covalent bond, only the second term should dominate. In this situation, one would expect from a purely electrostatic perspective, for *q*_*a*_ ∼ −0.1*e* and *d* ∼ 2 Å, a linear variation of the SOE with respect to the dopant charge with a slope of about 0.7 V. This is, of course, an oversimplified scenario, as the electronic structure of the adsorbate is not frozen and is affected by interactions with the surface. For instance, further Bader charge calculations on adsorbed states show that O takes 0.20*e* from Ni on NiAu and 0.32*e* from Pt on PtCu. Nevertheless, if the electrostatic term dominates, the dopant charge is likely to remain a good descriptor and the SOE should correlate with the dopant charge as predicted by eq iii. The qualitative agreement with the DFT data plotted in Figure 2 supports the electrostatic origin of the variation of the SOE. It is important to note, however, that the partitioning scheme used to define atomic charges plays a key role in the quality of the correlation. DDEC6 and HD charges do not quantitatively correlate with the SOEs (Figures S3 and S4). Therefore, we only consider charges obtained with the Bader analysis throughout the rest of this work.

**Figure 2:**
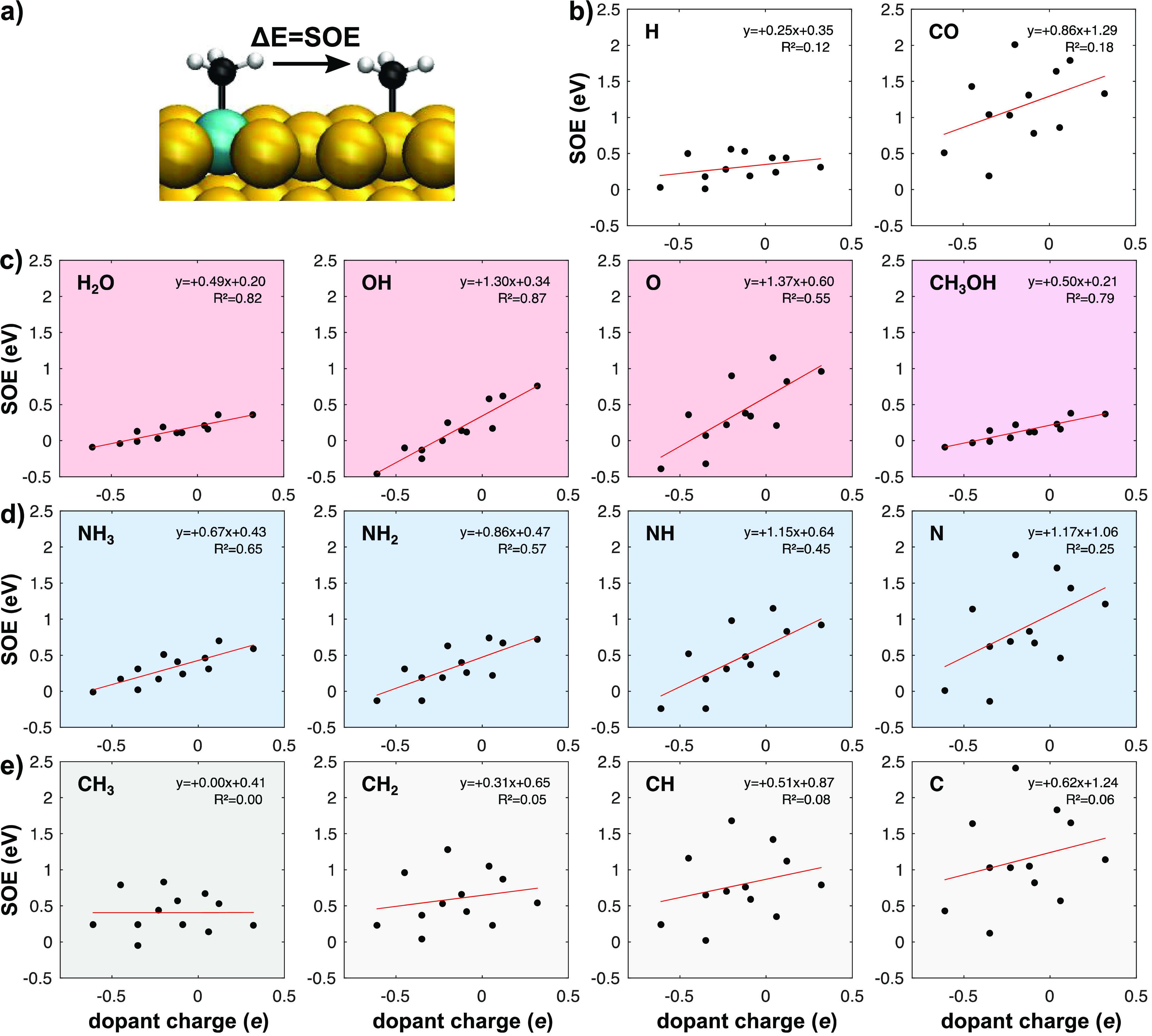
Correlation between the spillover energy (SOE) and the dopant charge for different adsorbates on 12 different SAAs. (a) The SOE is defined as the energy difference between the adsorbate (in this case methyl) bonding at the dopant site (cyan) and at a distant site on the host (yellow). (b–e) SOE plotted against the dopant charge (in units of the elementary charge *e*) for (b) H and CO as well as other absorbates bound via a (c) O, (d) N, and (e) C atom. For each adsorbate, the least-squares linear fit is plotted as a red line.

Analysis of the intercept of the linear correlations (Figure 2) is also insightful as it estimates an averaged covalent contribution to the SOE. Saturated species do not generally need to form extra chemical bonds as they are already stable. For these species, the covalent contribution Δ*E*_*cov*_ is expected to be small, making the electrostatic term dominant. When the degree of saturation decreases, the adsorbate’s orbitals tend to hybridize more with those of the surface, resulting in the formation of a chemical bond. In this situation, the covalent contribution Δ*E*_*cov*_ is expected to be larger as chemical bonds with transition metals tend to be stronger than with coinage metals (Table S2). The plots in Figure 2 are consistent with this analysis: the value of the intercept increases when the degree of saturation of the adsorbates decreases (Figure 2c–e). It is also important to note that Δ*E*_*cov*_ is a complicated function of the chemical nature of both the surface and the adsorbate. As expected, when Δ*E*_*cov*_ becomes dominant over Δ*E*_*elec*_, the SOE versus dopant charge plots become noticeably scattered. Indeed, the SOEs of unsaturated adsorbates (O, NH_2_, NH, N) and CH_*z*_, H, and CO (Figure 2b–e) are consistent with this analysis. For these adsorbates, the dopant charge is not a good descriptor for the SOE, and another descriptor must be found.

To this end, we have replotted the data as a function of the SOE of C, the species that shows the largest deviation in our previous attempt to use the dopant charge as a descriptor (Figure S5). Figure 3a provides a comparison between the quality of the regression of the SOEs against the dopant charge on the one hand (variable *x*), and the SOE of C on the other hand (variable *y*). Interestingly, the points of this plot lie close to the line *y* = 1 – *x*. In fact, from this plot, we can qualitatively classify the species on a scale that goes from predominantly covalent interactions (upper-left corner) to predominantly electrostatic contributions (lower-right corner) as shown in Figure 3b. This classification is consistent with chemical intuition when considering the degree of unsaturation of the species and their electronegativities (χ_*H*_ = 2.20, χ_*C*_ = 2.55, χ_*N*_ = 3.04, and χ_*O*_ = 3.44) compared with those of the dopants (1.91 ≤ χ_*d*_ ≤ 2.28).

**Figure 3:**
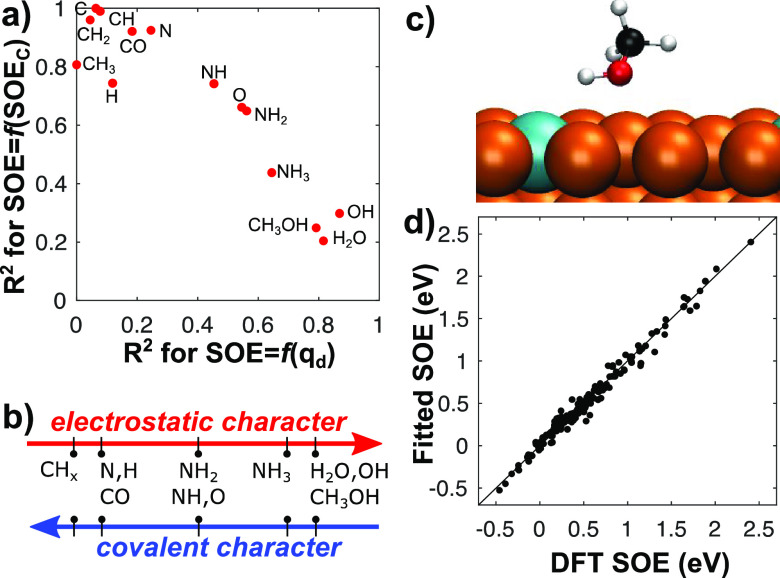
Bonding mechanism of adsorbates on SAAs. (a) Correlation coefficients of the two one-parameter models for each adsorbate. The *x*-axis gives the correlation coefficients for the model using only the dopant charge *q*_*d*_ as a parameter. The *y*-axis gives the correlation coefficients for the model using only the SOE of C as a parameter. (b) Classification of adsorbates with increasing electrostatic/covalent character in their bonding mechanism to SAA surfaces. (c) Unique adsorption geometry of CH_3_OH on PtCu(111) SAA. (d) Parity plot of the SOEs fitted on both the dopant charge and the SOE of C (two-parameter model) versus the DFT-computed SOEs.

The analysis of methanol adsorption on PtCu reveals a third type of interaction. On this SAA, the SOE of methanol, when bound to the dopant via O, is negative (−0.09 eV): methanol should therefore prefer to bind to host sites distant from the dopant site. However, another type of binding site was reported for alcohols where the molecule adsorbs on a Cu atom at the vicinity of Pt with the O–H bond pointing toward the dopant (Figure 3c).^31,46^ This geometry is more stable than on pure Cu host sites and brings the SOE of methanol up to +0.04 eV. A similar binding configuration was found for H_2_O on PtCu, bringing the SOE from −0.09 eV (for O on top of the dopant atom) to +0.02 eV (on top of the vicinal Cu site). This overstabilization is even more unexpected considering that, around the dopant, there exists an exclusion zone that destabilizes adsorbates with respect to distant host sites.^47^ Interestingly, this more stable binding configuration could only be identified on PtCu, where the dopant charge is the most negative among all SAAs investigated. This interaction can be analyzed as a monopole-dipole interaction or weak hydrogen bond and could lead to enhanced reactivity of polarized O–H bonds. This is in line with the DFT investigations of the mechanism of the dry dehydrogenation of ethanol to acetaldehyde. On PtCu (*q*_*d*_ = −0.61*e*), the O–H cleavage is reported to be the first elementary step,^46^ whereas on NiAu (*q*_*d*_ = +0.32*e*), the C–H cleavage occurs first.^39^

To build a quantitative surrogate model that simultaneously captures the effects of both bonding mechanisms, we have performed a multilinear regression using both the dopant charge and the SOE of C as descriptors. The parameters of the regression are given in Table S5. Figure 3d shows the parity plot of the fitted SOEs against the DFT-computed SOEs. The SOEs are reproduced with a mean absolute error (MAE) of 0.06 eV and a standard deviation of 0.07 eV. This excellent agreement confirms that the dopant charge and the SOE of C are good descriptors for the estimation of SOEs on SAAs. Although the interaction of C with metal surfaces is easy to compute using *ab initio* methods, it is more difficult to obtain from experimental data. There is, however, growing experimental data on the adsorption energies of H and CO on SAAs.^8,10,48^ We have therefore considered the SOEs of H and CO, both at the covalent end of the scale shown in Figure 3b, as alternatives for the covalent parameter of the surrogate model. The SOE of H turns out to be a poor descriptor of the nonelectrostatic contribution (Table S5). The SOE of CO, albeit not as good as the SOE of C, performs well with a MAE of 0.07 eV and a standard deviation of 0.10 eV. Thus, the SOE of CO could also be used, instead of the SOE of C, should the latter be unknown.

In conclusion, through a detailed study of common adsorbates on SAA surfaces, we have furthered the understanding of the relative binding of adsorbates to host and dopant sites. We have shown that the electronegativity difference between the dopant and the host atoms results in the formation of atomic charges localized on the dopant atom, with the countercharge fully delocalized on the host. Our work, therefore, provides theoretical rationalization of experimental observations suggesting the presence of charges located at the dopant of SAA surfaces. The dopant charge significantly affects the relative stability of chemisorbed species between dopant and host sites. Furthermore, the dopant charge alone is a quantitative descriptor for the SOE of saturated species bound to the surface via their lone pair (H_2_O, CH_3_OH, NH_3_). The dopant charge is also a qualitative descriptor for the SOEs of the catalytically relevant OH_*x*_ and NH_*y*_ chemical intermediates, which are more stable on host sites when the dopant carries a high negative charge. Including the SOE of C as a second regression parameter systematically improves the correlation for all the chemisorbed species considered in this study. The combination of these two descriptors captures, by disentangling the covalent and electrostatic contributions, the main bonding mechanisms of surface species on SAAs, regardless of whether the species are chemically related to C or not. In that sense, our charge-inclusive thermochemical scaling relationships offer more versatility and transversality on SAAs than traditional thermochemical scaling relationships, which tend to solely correlate the binding energies of chemically related fragments.^31,49^ Our model offers a simple guide in the design of new SAA catalysts based on the charge of the dopant metal atoms and their affinity for carbon. Finally, our work indicates that in the continued development of high-throughput and machine-learning models for alloy catalysts,^50−52^ it would be prudent to consider the local charge distribution of the clean alloy surfaces as input parameters, since they may outperform traditional descriptors.

## Supporting Information Available

Computational details, tables including charges (Bader, HD, and DDEC6) and SOEs, and parameters and accuracy of the fitted model (PDF)
Transparent Peer Review report available (PDF)
Optimized structures (ZIP)

## References

[ref1] Hannagan R. T.; Giannakakis G.; Flytzani-Stephanopoulos M.; Sykes E. C. H. Single-Atom Alloy Catalysis. Chem. Rev. 2020, 120 (21), 12044–12088. 10.1021/acs.chemrev.0c00078. 32588624

[ref2] Réocreux R.; Stamatakis M. One Decade of Computational Studies on Single-Atom Alloys: Is In Silico Design within Reach?. Acc. Chem. Res. 2022, 55 (1), 87–97. 10.1021/acs.accounts.1c00611. 34904820

[ref3] Zhang T.; Walsh A. G.; Yu J.; Zhang P. Single-Atom Alloy Catalysts: Structural Analysis, Electronic Properties and Catalytic Activities. Chem. Soc. Rev. 2021, 50 (1), 569–588. 10.1039/D0CS00844C. 33170202

[ref4] Hannagan R. T.; Giannakakis G.; Réocreux R.; Schumann J.; Finzel J.; Wang Y.; Michaelides A.; Deshlahra P.; Christopher P.; Flytzani-Stephanopoulos M.; Stamatakis M.; Sykes E. C. H. First-Principles Design of a Single-Atom–Alloy Propane Dehydrogenation Catalyst. Science 2021, 372 (6549), 1444–1447. 10.1126/science.abg8389.

[ref5] Zhou L.; Martirez J. M. P.; Finzel J.; Zhang C.; Swearer D. F.; Tian S.; Robatjazi H.; Lou M.; Dong L.; Henderson L.; Christopher P.; Carter E. A.; Nordlander P.; Halas N. J. Light-Driven Methane Dry Reforming with Single Atomic Site Antenna-Reactor Plasmonic Photocatalysts. Nat. Energy 2020, 5 (1), 61–70. 10.1038/s41560-019-0517-9.

[ref6] Tierney H. L.; Baber A. E.; Kitchin J. R.; Sykes E. C. H. Hydrogen Dissociation and Spillover on Individual Isolated Palladium Atoms. Phys. Rev. Lett. 2009, 103 (24), 246102 10.1103/PhysRevLett.103.246102. 20366214

[ref7] Kyriakou G.; Boucher M. B.; Jewell A. D.; Lewis E. A.; Lawton T. J.; Baber A. E.; Tierney H. L.; Flytzani-Stephanopoulos M.; Sykes E. C. H. Isolated Metal Atom Geometries as a Strategy for Selective Heterogeneous Hydrogenations. Science 2012, 335 (6073), 1209–1212. 10.1126/science.1215864. 22403387

[ref8] Darby M. T.; Lucci F. R.; Marcinkowski M. D.; Therrien A. J.; Michaelides A.; Stamatakis M.; Sykes E. C. H. Carbon Monoxide Mediated Hydrogen Release from PtCu Single-Atom Alloys: The Punctured Molecular Cork Effect. J. Phys. Chem. C 2019, 123 (16), 10419–10428. 10.1021/acs.jpcc.9b01213.

[ref9] Kress P.; Réocreux R.; Hannagan R.; Thuening T.; Boscoboinik J. A.; Stamatakis M.; Sykes E. C. H. Mechanistic Insights into Carbon–Carbon Coupling on NiAu and PdAu Single-Atom Alloys. J. Chem. Phys. 2021, 154 (20), 204701 10.1063/5.0048977. 34241183

[ref10] Darby M. T.; Sykes E. C. H.; Michaelides A.; Stamatakis M. Carbon Monoxide Poisoning Resistance and Structural Stability of Single Atom Alloys. Top. Catal. 2018, 61 (5–6), 428–438. 10.1007/s11244-017-0882-1. 31258304 PMC6560695

[ref11] Liu M.; Yang Y.; Kitchin J. R. Semi-Grand Canonical Monte Carlo Simulation of the Acrolein Induced Surface Segregation and Aggregation of AgPd with Machine Learning Surrogate Models. J. Chem. Phys. 2021, 154 (13), 134701 10.1063/5.0046440. 33832264

[ref12] Finzel J.; Christopher P. Dynamic Pt Coordination in Dilute AgPt Alloy Nanoparticle Catalysts Under Reactive Environments. Top. Catal. 2022, 10.1007/s11244-021-01545-7.

[ref13] Wang Q.; Zhu B.; Tielens F.; Tichit D.; Guesmi H. Mapping Surface Segregation of Single-Atom Pt Dispersed in M Surfaces (M = Cu, Ag, Au, Ni, Pd, Co, Rh and Ir) under Hydrogen Pressure at Various Temperatures. Appl. Surf. Sci. 2021, 548, 149217 10.1016/j.apsusc.2021.149217.

[ref14] Han Z. K.; Sarker D.; Ouyang R.; Mazheika A.; Gao Y.; Levchenko S. V. Single-Atom Alloy Catalysts Designed by First-Principles Calculations and Artificial Intelligence. Nat. Commun. 2021, 12, 1833 10.1038/s41467-021-22048-9. 33758170 PMC7988173

[ref15] Wang D.; Cao R.; Hao S.; Liang C.; Chen G.; Chen P.; Li Y.; Zou X. Accelerated Prediction of Cu-Based Single-Atom Alloy Catalysts for CO2 Reduction by Machine Learning. Green Energy Environ. 2021, 10.1016/j.gee.2021.10.003.

[ref16] Kumar A.; Iyer J.; Jalid F.; Ramteke M.; Khan T. S.; Haider M. A. Machine Learning Enabled Screening of Single Atom Alloys: Predicting Reactivity Trend for Ethanol Dehydrogenation. ChemCatChem. 2022, 14 (2), 1–13. 10.1002/cctc.202101481.

[ref17] Zheng G.; Li Y.; Qian X.; Yao G.; Tian Z.; Zhang X.; Chen L. High-Throughput Screening of a Single-Atom Alloy for Electroreduction of Dinitrogen to Ammonia. ACS Appl. Mater. Interfaces 2021, 13 (14), 16336–16344. 10.1021/acsami.1c01098. 33797214

[ref18] Sun Z.; Song Z.; Yin W.-J. Going Beyond the D-Band Center to Describe CO2 Activation on Single-Atom Alloys. Adv. Energy Sustain. Res. 2022, 3 (2), 2100152 10.1002/aesr.202100152.

[ref19] Abild-Pedersen F.; Greeley J.; Studt F.; Rossmeisl J.; Munter T. R.; Moses P. G.; Skúlason E.; Bligaard T.; Nørskov J. K. Scaling Properties of Adsorption Energies for Hydrogen-Containing Molecules on Transition-Metal Surfaces. Phys. Rev. Lett. 2007, 99, 016105 10.1103/PhysRevLett.99.016105. 17678168

[ref20] Liu Z.-P.; Hu P. General Trends in the Barriers of Catalytic Reactions on Transition Metal Surfaces. J. Chem. Phys. 2001, 115 (11), 4977–4980. 10.1063/1.1403006.

[ref21] Michaelides A.; Liu Z.-P.; Zhang C. J.; Alavi A.; King D. A.; Hu P. Identification of General Linear Relationships between Activation Energies and Enthalpy Changes for Dissociation Reactions at Surfaces. J. Am. Chem. Soc. 2003, 125 (13), 3704–3705. 10.1021/ja027366r. 12656593

[ref22] Nørskov J. K.; Bligaard T.; Rossmeisl J.; Christensen C. H. Towards the Computational Design of Solid Catalysts. Nat. Chem. 2009, 1 (1), 37–46. 10.1038/nchem.121. 21378799

[ref23] Nørsko J. K. Chemisorption on Metal Surfaces. Rep. Prog. Phys. 1990, 53 (10), 1253–1295. 10.1088/0034-4885/53/10/001.

[ref24] Hammer B.; Norskov J. K. Why Gold Is the Noblest of All the Metals. Nature 1995, 376 (6537), 238–240. 10.1038/376238a0.

[ref25] Calle-Vallejo F.; Martínez J. I.; García-Lastra J. M.; Sautet P.; Loffreda D. Fast Prediction of Adsorption Properties for Platinum Nanocatalysts with Generalized Coordination Numbers. Angew. Chemie Int. Ed. 2014, 53 (32), 8316–8319. 10.1002/anie.201402958. 24919964

[ref26] Nørskov J. K.; Bligaard T.; Rossmeisl J.; Christensen C. H. Towards the Computational Design of Solid Catalysts. Nat. Chem. 2009, 1 (1), 37–46. 10.1038/nchem.121. 21378799

[ref27] Calle-Vallejo F.; Tymoczko J.; Colic V.; Vu Q. H.; Pohl M. D.; Morgenstern K.; Loffreda D.; Sautet P.; Schuhmann W.; Bandarenka A. S. Finding Optimal Surface Sites on Heterogeneous Catalysts by Counting Nearest Neighbors. Science 2015, 350 (6257), 185–189. 10.1126/science.aab3501. 26450207

[ref28] Hensley A. J. R.; Wang Y.; McEwen J.-S. Adsorption of Guaiacol on Fe (110) and Pd (111) from First Principles. Surf. Sci. 2016, 648, 227–235. 10.1016/j.susc.2015.10.030.

[ref29] Jørgensen M.; Grönbeck H. Scaling Relations and Kinetic Monte Carlo Simulations To Bridge the Materials Gap in Heterogeneous Catalysis. ACS Catal. 2017, 7 (8), 5054–5061. 10.1021/acscatal.7b01194.

[ref30] Thirumalai H.; Kitchin J. R. Investigating the Reactivity of Single Atom Alloys Using Density Functional Theory. Top. Catal. 2018, 61 (5–6), 462–474. 10.1007/s11244-018-0899-0.

[ref31] Darby M. T.; Réocreux R.; Sykes E. C. H.; Michaelides A.; Stamatakis M. Elucidating the Stability and Reactivity of Surface Intermediates on Single-Atom Alloy Catalysts. ACS Catal. 2018, 8 (6), 5038–5050. 10.1021/acscatal.8b00881.

[ref32] Gao D.; Yi D.; Lu F.; Li S.; Pan L.; Xu Y.; Wang X. Orbital-Scale Understanding on High-Selective Hydrogenation of Acetylene over Pt1-Cu(111) Catalyst. Chem. Eng. Sci. 2021, 240, 116664 10.1016/j.ces.2021.116664.

[ref33] Spivey T. D.; Holewinski A. Selective Interactions between Free-Atom-like d-States in Single-Atom Alloy Catalysts and Near-Frontier Molecular Orbitals. J. Am. Chem. Soc. 2021, 143 (31), 11897–11902. 10.1021/jacs.1c04234. 34319717

[ref34] Greiner M. T.; Jones T. E.; Beeg S.; Zwiener L.; Scherzer M.; Girgsdies F.; Piccinin S.; Armbrüster M.; Knop-Gericke A.; Schlögl R. Free-Atom-like d-States in Single-Atom Alloy Catalysts. Nat. Chem. 2018, 10, 1008–1015. 10.1038/s41557-018-0125-5. 30150725

[ref35] Zhao G.-C.; Qiu Y.-Q.; Liu C.-G. A Systematic Theoretical Study of Hydrogen Activation, Spillover and Desorption in Single-Atom Alloys. Appl. Catal. A Gen. 2021, 610, 117948 10.1016/j.apcata.2020.117948.

[ref36] Shi J.; Owen C. J.; Ngan H. T.; Qin S.; Mehar V.; Sautet P.; Weaver J. F. Formation of a Ti–Cu(111) Single Atom Alloy: Structure and CO Binding. J. Chem. Phys. 2021, 154 (23), 234703 10.1063/5.0050800. 34241242

[ref37] Fako E.; Łodziana Z.; López N. Comparative Single Atom Heterogeneous Catalysts (SAHCs) on Different Platforms: A Theoretical Approach. Catal. Sci. Technol. 2017, 7 (19), 4285–4293. 10.1039/C7CY01136A.

[ref38] Chen C.; Wu D.; Li Z.; Zhang R.; Kuai C.; Zhao X.; Dong C.; Qiao S.; Liu H.; Du X. Ruthenium-Based Single-Atom Alloy with High Electrocatalytic Activity for Hydrogen Evolution. Adv. Energy Mater. 2019, 9 (20), 1803913 10.1002/aenm.201803913.

[ref39] Giannakakis G.; Kress P.; Duanmu K.; Ngan H. T.; Yan G.; Hoffman A. S.; Qi Z.; Trimpalis A.; Annamalai L.; Ouyang M.; Liu J.; Eagan N.; Biener J.; Sokaras D.; Flytzani-Stephanopoulos M.; Bare S. R.; Sautet P.; Sykes E. C. H. Mechanistic and Electronic Insights into a Working NiAu Single-Atom Alloy Ethanol Dehydrogenation Catalyst. J. Am. Chem. Soc. 2021, 143 (51), 21567–21579. 10.1021/jacs.1c09274. 34908398

[ref40] Klimeš J.; Bowler D. R.; Michaelides A. Van Der Waals Density Functionals Applied to Solids. Phys. Rev. B - Condens. Matter Mater. Phys. 2011, 83 (19), 1–13. 10.1103/PhysRevB.83.195131.

[ref41] Bader R. F. W. Atoms in Molecules: A Quantum Theory; Oxford University Press: Oxford, U.K., 1994.

[ref42] Manz T. A.; Limas N. G. Introducing DDEC6 Atomic Population Analysis: Part 1. Charge Partitioning Theory and Methodology. RSC Adv. 2016, 6 (53), 47771–47801. 10.1039/C6RA04656H. PMC9096813 35703680

[ref43] Limas N. G.; Manz T. A. Introducing DDEC6 Atomic Population Analysis: Part 2. Computed Results for a Wide Range of Periodic and Nonperiodic Materials. RSC Adv. 2016, 6 (51), 45727–45747. 10.1039/C6RA05507A.

[ref44] Hirshfeld F. L. Bonded-Atom Fragments for Describing Molecular Charge Densities. Theor. Chim. Acta 1977, 44 (2), 129–138. 10.1007/BF00549096.

[ref45] Perdew J. P.; Burke K.; Ernzerhof M. Generalized Gradient Approximation Made Simple. Phys. Rev. Lett. 1996, 77 (18), 3865–3868. 10.1103/PhysRevLett.77.3865. 10062328

[ref46] Wang Z.-T.; Hoyt R. A.; El-Soda M.; Madix R. J.; Kaxiras E.; Sykes E. C. H. Dry Dehydrogenation of Ethanol on Pt–Cu Single Atom Alloys. Top. Catal. 2018, 61 (5–6), 328–335. 10.1007/s11244-017-0856-3.

[ref47] Schumann J.; Bao Y.; Hannagan R. T.; Sykes E. C. H.; Stamatakis M.; Michaelides A. Periodic Trends in Adsorption Energies around Single-Atom Alloy Active Sites. J. Phys. Chem. Lett. 2021, 12 (41), 10060–10067. 10.1021/acs.jpclett.1c02497. 34632767

[ref48] Liu J.; Lucci F. R.; Yang M.; Lee S.; Marcinkowski M. D.; Therrien A. J.; Williams C. T.; Sykes E. C. H.; Flytzani-Stephanopoulos M. Tackling CO Poisoning with Single-Atom Alloy Catalysts. J. Am. Chem. Soc. 2016, 138 (20), 6396–6399. 10.1021/jacs.6b03339. 27167705

[ref49] Vijay S.; Kastlunger G.; Chan K.; Nørskov J. K. Limits to Scaling Relations between Adsorption Energies. J. Chem. Phys. 2022, 156 (23), 231102 10.1063/5.0096625. 35732521

[ref50] Kitchin J. R. Machine Learning in Catalysis. Nat. Catal. 2018, 1 (4), 230–232. 10.1038/s41929-018-0056-y.

[ref51] Andersen M.; Levchenko S. V.; Scheffler M.; Reuter K. Beyond Scaling Relations for the Description of Catalytic Materials. ACS Catal. 2019, 9 (4), 2752–2759. 10.1021/acscatal.8b04478.

[ref52] García-Muelas R.; López N. Statistical Learning Goes beyond the D-Band Model Providing the Thermochemistry of Adsorbates on Transition Metals. Nat. Commun. 2019, 10 (1), 4687 10.1038/s41467-019-12709-1. 31615991 PMC6794282

