# Supplementary material for: Stick or Spill? Scaling Relationships for the Binding Energies of Adsorbates on Single-Atom Alloy Catalysts
Source: J Phys Chem Lett. 2022 Aug 2;13(31):7314–9. doi: 10.1021/acs.jpclett.2c01519 (PMC9376958; doi:10.1021/acs.jpclett.2c01519)
Supplement: Supplementary file 1 — jz2c01519_si_001.pdf [file jz2c01519_si_001.pdf]

SUPPLEMENTARY INFORMATION

Stick or Spill? Scaling Relationships for the Binding Energies of Adsorbates on Single-Atom Alloy Catalysts

R. Réocreux,[1]* E.C.H. Sykes,[2] A. Michaelides,[3] M. Stamatakis[1]*
romain.reocreux@gmail.com, m.stamatakis@ucl.ac.uk

[1] Thomas Young Centre and Department of Chemical Engineering, University College London, Roberts Building, Torrington Place, London WC1E 7JE, UK.
[2] Department of Chemistry, Tufts University, Medford, MA 02155, USA.
[3] Yusuf Hamied Department of Chemistry, University of Cambridge, Lensfield Road, CB2 1EW Cambridge, UK.

Role of spill-over entropy.

Considering top sites only, the spillover entropy (from dopant, with surface loading δ, to host sites) can be approximated by the following formula:

$$\Delta_{SO}S = -k_B \ln\left(\frac{\delta}{1-\delta}\right)$$

For typical dopant loadings of $\delta = 5\%$ to $\delta = 0.1\%$, the spillover entropy can range from 24 to 57 $J{\cdot}mol^{-1}{\cdot}K^{-1}$. At room temperature, this translates into a $T\Delta_{SO}S$ contribution to the spillover free energy that ranges from 0.07 eV to 0.18 eV. This contribution only plays a role for small SOEs and becomes more significant at higher temperatures or for very low dopant concentrations (unlike the SOE). For instance, the SOEs of C range from 0.12 eV to 2.41 eV.

Computational details.

Spillover Energy calculations.

The DFT calculations were performed using the Vienna *Ab Initio* Simulation Package (VASP) version 5.4.4.[1–3] The non-local optB86b-vdW functional was used to describe the exchange-correlation potential.[4–6] The core electrons were treated using the Projected Augmented wave (PAW) method.[7,8] A plane wave basis set truncated at 400 eV was used to expand the valence density. The electronic energies were converged to 10^{-7} eV. The lattice constants used in this work were taken from our previous work (Au: 4.127 Å, Ag: 4.077 Å, Cu: 3.608 Å).[9] Five-layer *p*(3×3) slabs were considered. The integration over the Brillouin zone was performed on a 13×13×1 Monkhorst-Pack k-point mesh.[10] Geometries were relaxed, freezing the atoms of the two bottom layers at their bulk positions. Complementary calculations for the Spill-Over Energy of OH were performed using the PBE functional with reoptimized lattice constants (Au: 4.172 Å, Ag: 4.165 Å, Cu: 3.635 Å) and otherwise-identical parameters.[11] All adsorbates were considered at their most stable adsorption sites. Unless stated otherwise, most adsorbates shared the same adsorption sites among the different SAA surfaces:

- fcc threefold sites for C, CH, H (top site on PtAg and PtAu), NH, O, OH (top site on RhAu)
- bridge sites for CH_2 (threefold site on NiCu, PdCu and PtCu) and NH_2
- top sites for CH_3 (threefold site on NiCu), NH_3, H_2O, CO, CH_3OH.

The optimized structures are provided as a zip file.

Atomic charge calculations.

Atomic charges were computed after reoptimizing the structure of the clean slabs using a larger basis set and a denser FFT grid. Increasing the basis-set cut-off with the `PREC=Accurate` flag automatically increases the density of the FFT grid. A cut-off energy of 500 eV was found to be necessary in the case of PtCu to converge

Bader charges to $10^{-2}e$. This set-up was used for all the SAA surfaces considered in this study. The Bader charge analysis was performed using the code developed by Henkelman and co-workers, available at http://theory.cm.utexas.edu/henkelman/code/bader/ .[12–15] Two other partitioning schemes were considered, namely the refined version of the Density Derived Electrostatic and Chemical approach (DDEC6) and the Hirshfeld-Dominant method (HD).[16] The DDEC6 charges were determined using CHARGEMOL,[17,18] a post-processing program available at https://sourceforge.net/projects/ddec. PBE+dDsC calculations were performed to calculate the HD charges. For these calculations the bulk structures were first reoptimized to determine the pertinent lattice constants (lattice constants: Au 4.118 Å, Ag 4.094 Å, Cu 3.591 Å).[11,19,20]

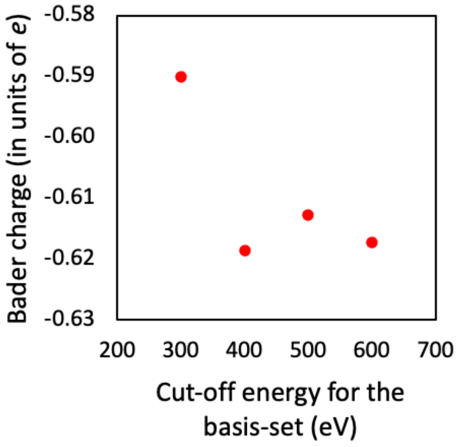

Figure S1. Convergence analysis of the Bader charge of Pt in PtCu SAA.

Supplementary Data.

Table S1. Atomic charges (Bader charges) on 12 different SAA surfaces: q_d is the dopant charge (in units of e, the elementary charge), $min(q_h)$ and $max(q_h)$ represent the smallest and the largest charges identified on the host material.

SAAs	*Bader charges*			*HD charges*	*DDEC6 charges*
	q_d	$max(q_h)$	$min(q_h)$	q_d	q_d
NiCu	-0.09	0.04	-0.03	-0.10	-0.15
NiAg	0.04	0.03	-0.04	-0.10	-0.14
NiAu	0.32	0.04	-0.08	-0.03	0.01
PdCu	-0.35	0.05	-0.03	-0.16	0.01
PdAg	-0.23	0.03	-0.03	-0.20	-0.06
PdAu	0.06	0.04	-0.05	-0.13	0.06
PtCu	-0.61	0.07	-0.03	-0.19	-0.23
PtAg	-0.45	0.05	-0.03	-0.23	-0.29
PtAu	-0.12	0.04	-0.04	-0.14	-0.09
RhCu	-0.35	0.05	-0.03	-0.20	-0.24
RhAg	-0.2	0.04	-0.03	-0.23	-0.27
RhAu	0.12	0.04	-0.06	-0.15	-0.08
$max(q_d)$	0.32			-0.03	0.06
$min(q_d)$	-0.61			-0.23	-0.29
$max(q_h)-min(q_d)$	0.93			0.20	0.35

Table S2. Pauling's electronegativities χ for the elements of interest.[21]

elements	H	C	N	O	Ni	Rh	Pd	Pt	Cu	Ag	Au
χ	2.20	2.55	3.04	3.44	1.91	2.28	2.20	2.28	1.90	1.93	2.54

Table S3. Formation energies of fragments adsorbed to the most favored adsorption site on various metal surfaces. The energies are referenced with respect to CH_4(g), NH_3(g), H_2(g), CO(g) and clean metal surfaces.

Surfaces	CH_3	CH_2	CH	C	NH_3	NH_2	NH	N	H_2O	OH	O	H	CO	CH_3OH
**Cu**	0.68	1.72	2.37	3.79	-0.76	-0.06	0.61	2.06	-1.41	-1.25	-0.43	-0.26	0.68	-0.43
**Ag**	1.19	2.66	3.70	5.24	-0.58	0.57	1.89	3.68	-1.34	-0.68	0.80	0.16	1.40	-0.35
**Au**	0.86	2.19	2.87	4.31	-0.65	0.72	1.87	3.36	-1.33	-0.05	1.19	0.17	1.31	-0.36
**NiCu**	0.44	1.30	1.79	2.97	-1.00	-0.32	0.24	1.38	-1.51	-1.37	-0.77	-0.44	-0.10	-0.54
**NiAg**	0.52	1.61	2.27	3.41	-1.03	-0.17	0.74	1.96	-1.55	-1.26	-0.35	-0.28	-0.25	-0.58
**NiAu**	0.63	1.65	2.08	3.17	-1.24	0.01	0.95	2.15	-1.70	-0.80	0.22	-0.14	-0.02	-0.74
**PdCu**	0.73	1.67	2.36	3.67	-0.78	0.07	0.85	2.20	-1.40	-1.00	-0.11	-0.27	0.49	-0.41
**PdAg**	0.75	2.12	3.00	4.21	-0.75	0.38	1.58	2.99	-1.37	-0.68	0.59	-0.12	0.37	-0.39
**PdAu**	0.71	1.96	2.52	3.75	-0.96	0.50	1.62	2.90	-1.49	-0.22	0.98	-0.07	0.46	-0.52
**PtCu**	0.44	1.48	2.13	3.36	-0.75	0.07	0.85	2.05	-1.32	-0.79	-0.04	-0.29	0.17	-0.34
**PtAg**	0.40	1.70	2.53	3.60	-0.74	0.27	1.37	2.54	-1.30	-0.58	0.44	-0.34	-0.03	-0.32
**PtAu**	0.29	1.53	2.11	3.26	-1.05	0.32	1.39	2.52	-1.44	-0.19	0.81	-0.36	0.00	-0.49
**RhCu**	0.44	1.35	1.72	2.76	-1.07	-0.25	0.44	1.44	-1.53	-1.12	-0.51	-0.44	-0.36	-0.57
**RhAg**	0.36	1.38	2.02	2.84	-1.09	-0.06	0.92	1.79	-1.53	-0.93	-0.10	-0.40	-0.61	-0.57
**RhAu**	0.33	1.32	1.75	2.67	-1.34	0.05	1.04	1.92	-1.70	-0.67	0.37	-0.27	-0.47	-0.75

Table S4. Spill-over energies (SOEs) of fragments adsorbed on the most favored adsorption site on SAA surfaces. The last column (OH^{pbe}) refers to the SOEs of OH evaluated with the PBE functional. The rest of the table is obtained from optB86b-vdW calculations. The absolute deviations between the two functionals are less than 0.04 eV. SOEs ≤ 0 are highlighted in red.

Surfaces	CH_3	CH_2	CH	C	NH_3	NH_2	NH	N	H_2O	OH	O	H	CO	CH_3OH	OH^{pbe}
**NiCu**	0.24	0.42	0.59	0.82	0.24	0.26	0.37	0.67	0.11	0.12	0.34	0.19	0.78	0.12	0.12
**NiAg**	0.67	1.05	1.42	1.83	0.46	0.74	1.15	1.71	0.21	0.58	1.15	0.44	1.64	0.23	0.55
**NiAu**	0.23	0.54	0.79	1.14	0.59	0.72	0.92	1.21	0.36	0.76	0.96	0.31	1.33	0.37	0.73
**PdCu**	-0.05	0.04	0.02	0.12	0.02	-0.13	-0.24	-0.14	-0.01	-0.25	-0.32	0.01	0.19	-0.01	-0.24
**PdAg**	0.44	0.53	0.70	1.03	0.17	0.19	0.31	0.69	0.03	0.00	0.22	0.28	1.03	0.04	-0.03
**PdAu**	0.14	0.23	0.35	0.57	0.31	0.22	0.24	0.46	0.16	0.17	0.21	0.24	0.86	0.16	0.15
**PtCu**	0.24	0.23	0.24	0.43	-0.01	-0.13	-0.24	0.01	-0.09	-0.46	-0.39	0.03	0.51	-0.09	-0.46
**PtAg**	0.79	0.96	1.16	1.64	0.17	0.31	0.52	1.14	-0.04	-0.10	0.36	0.50	1.43	-0.03	-0.12
**PtAu**	0.57	0.66	0.76	1.05	0.41	0.40	0.48	0.83	0.11	0.14	0.38	0.53	1.31	0.12	0.13
**RhCu**	0.24	0.37	0.65	1.03	0.31	0.19	0.17	0.62	0.13	-0.13	0.07	0.18	1.04	0.14	-0.13
**RhAg**	0.83	1.28	1.68	2.41	0.51	0.63	0.98	1.89	0.19	0.25	0.90	0.56	2.01	0.22	0.21
**RhAu**	0.53	0.87	1.12	1.65	0.70	0.67	0.83	1.43	0.36	0.62	0.82	0.44	1.79	0.38	0.60

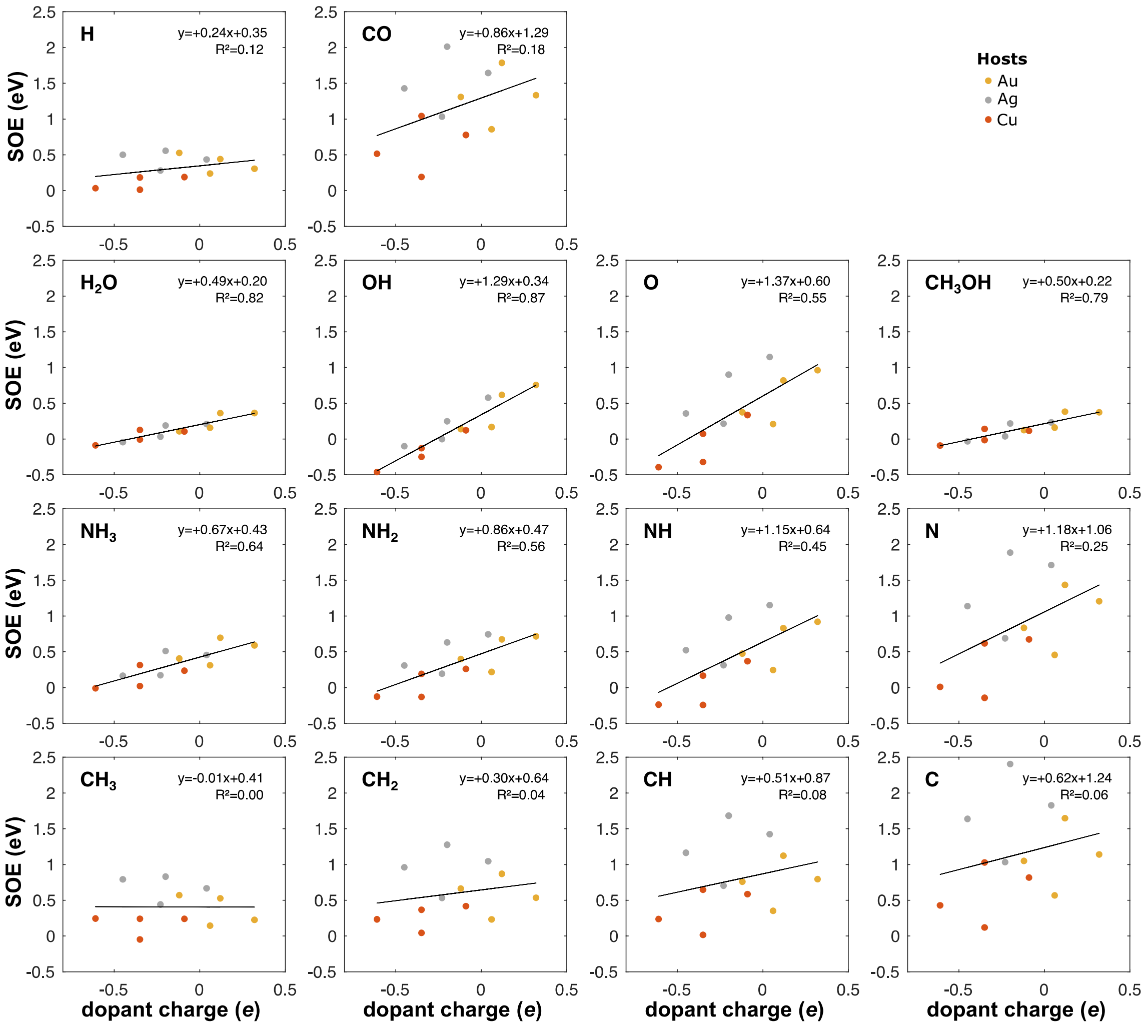

Figure S2. Regression of the SOEs against the dopant charge (Bader approach). The line provides the least-squares linear fit. This is the same data as plotted in Figure 2, focusing on the host material rather than the nature of the adsorbates.

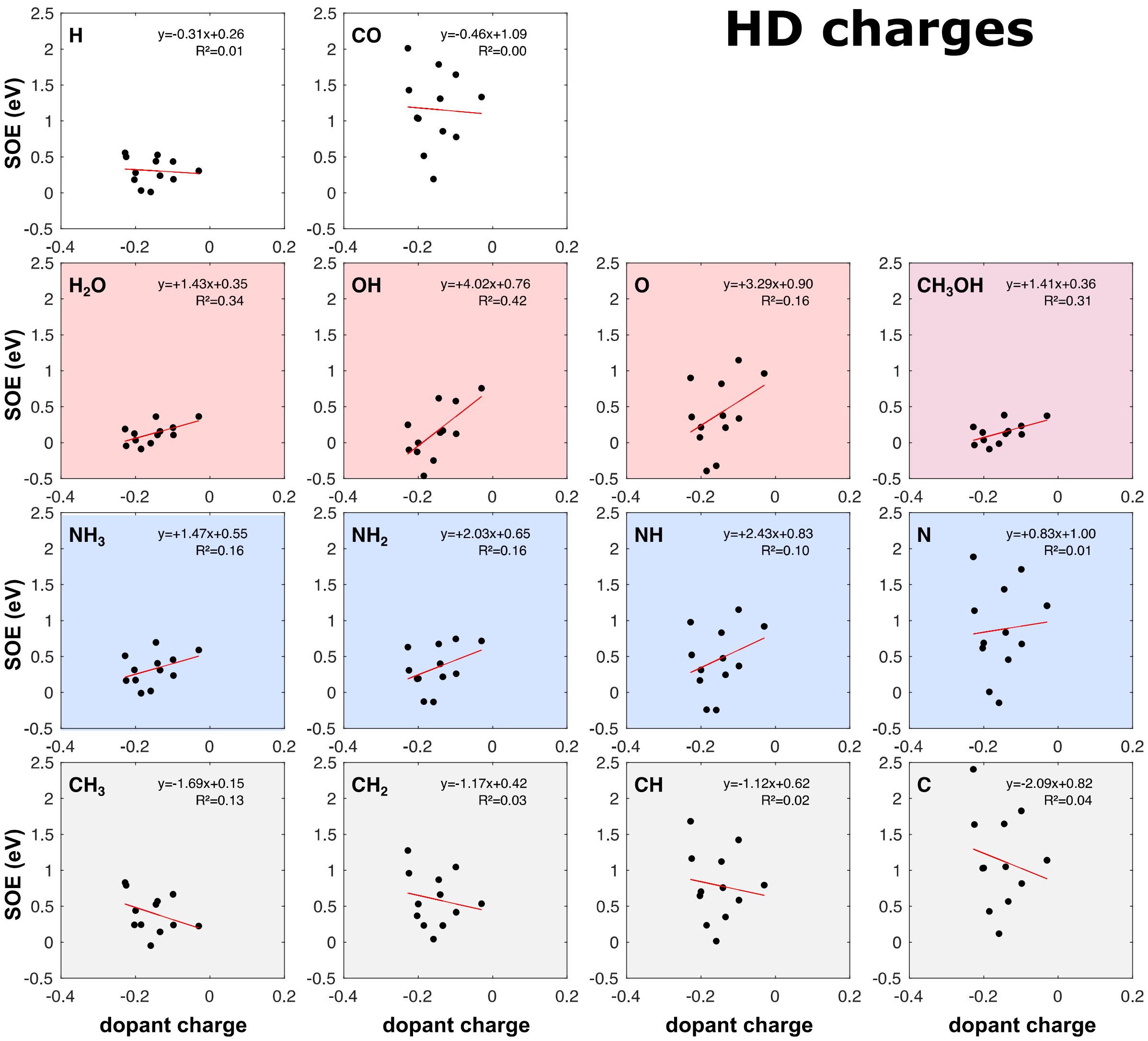

Figure S3. Regression of the SOEs against the HD charges. The red line provides the least-squares linear fit. Correlations are very poor with correlation coefficients ≤ 0.42.

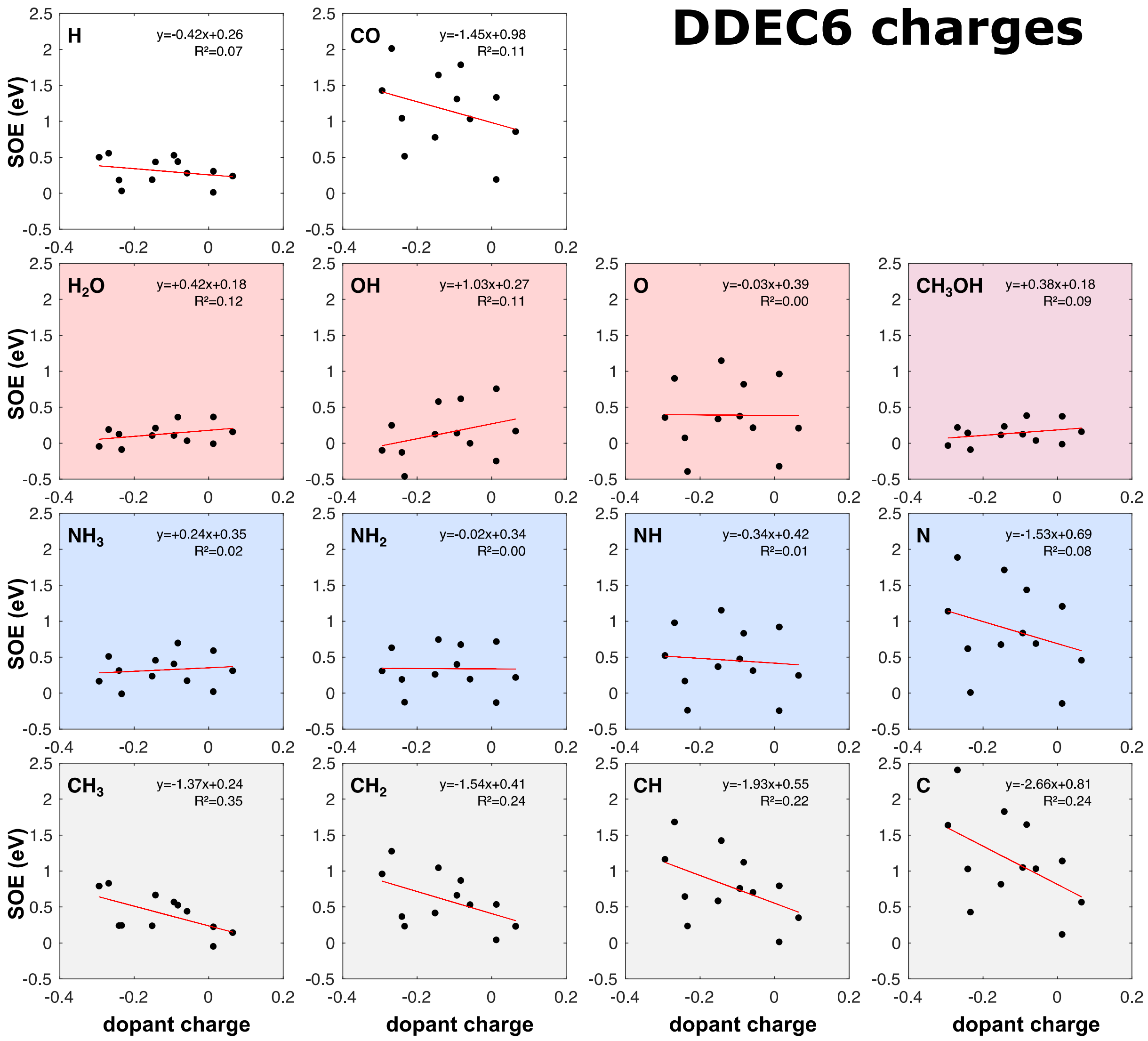

Figure S4. Regression of the SOEs against the DDEC6 charges. The red line provides the least-squares linear fit. Correlations are very poor with correlation coefficients ≤ 0.35.

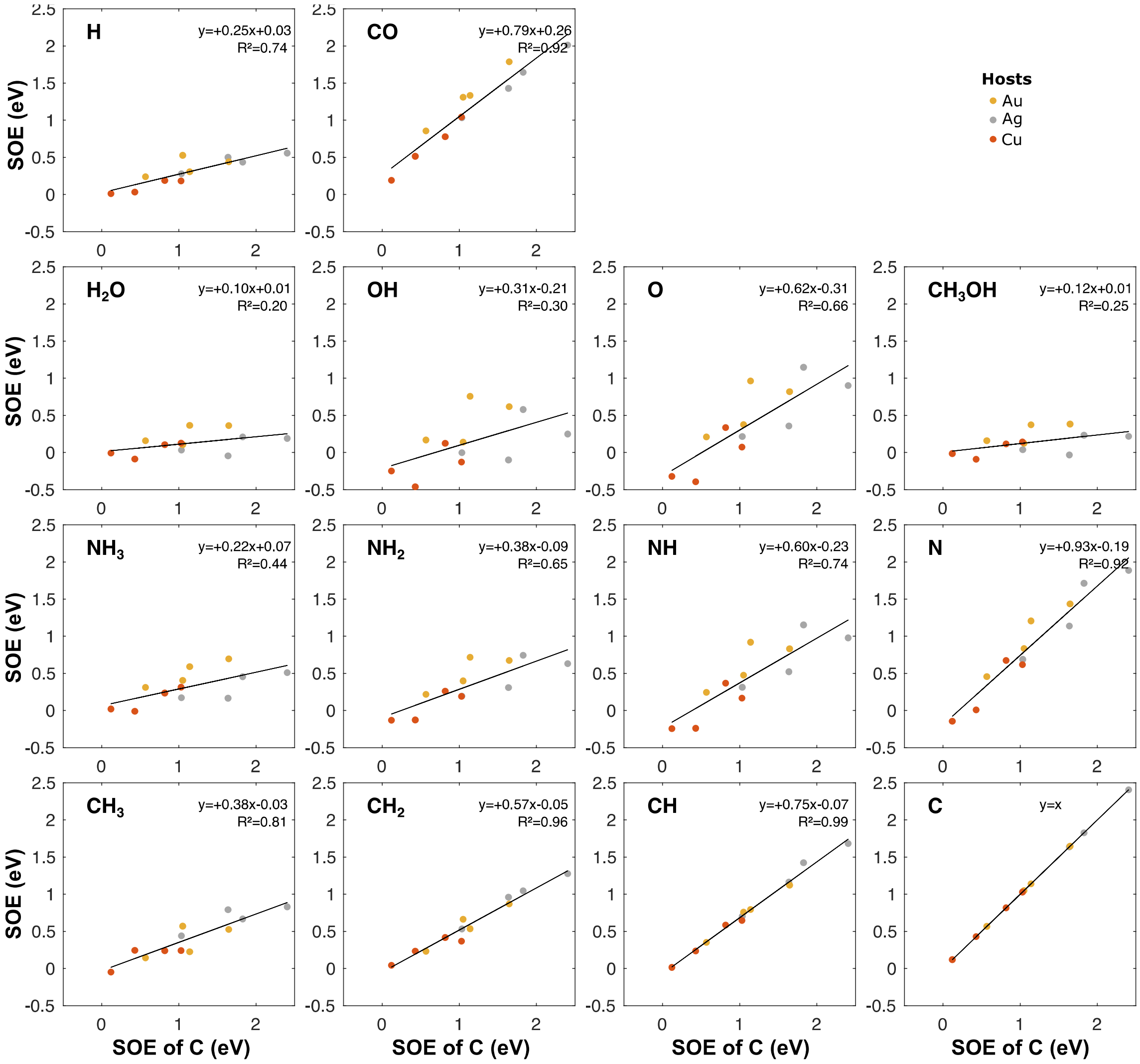

Figure S5. Regression of the SOEs against the SOE of C. The black line provides the least-squares linear fit.

Table S5. Parameters of the multilinear regression $SOE = \alpha + \beta \times q_d + \gamma \times SOE_k$ with $k \in \{H, CO, C\}$. MIN, MAX, MAE and STD stand for the minimum and maximum deviations, the mean absolute error, and the standard deviation. MAE and STD are not reported for the adsorbate whose SOE is used as a descriptor.

**adsorbates**	**Parameters and Accuracy of the surrogate model**						
**k = H**	**α (eV)**	**β (V)**	**γ**	**MIN**	**MAX**	**MAE**	**STD**
CH_3	-0.1154	-0.3705	1.5045	-0.1425	0.1523	0.0621	0.0805
CH_2	-0.0097	-0.1583	1.8901	-0.2395	0.3432	0.1108	0.1581
CH	0.0702	-0.0457	2.3113	-0.3499	0.5354	0.1687	0.2392
C	0.1784	-0.1243	3.0598	-0.4974	0.7574	0.2320	0.3256
NH_3	0.2453	0.5398	0.5200	-0.1620	0.0973	0.0787	0.0941
NH_2	0.1391	0.6258	0.9641	-0.1602	0.1895	0.0891	0.1096
NH	0.0840	0.7658	1.5939	-0.3439	0.3578	0.1345	0.1853
N	0.1427	0.5345	2.6453	-0.3976	0.6393	0.2139	0.2896
H_2O	0.1719	0.4692	0.0887	-0.1026	0.0631	0.0499	0.0597
OH	0.1598	1.1645	0.5242	-0.1444	0.1868	0.0703	0.0959
O	0.0806	1.0022	1.5072	-0.3708	0.3799	0.1448	0.1992
H	0	0	1	–	–	–	–
CO	0.4356	0.2591	2.4811	-0.2464	0.4033	0.1747	0.2078
CH_3OH	0.1685	0.4711	0.1360	-0.1137	0.0696	0.0528	0.0641
all but H	–	–	–	*-0.4974*	*0.7574*	*0.1217*	*0.1754*
**k = CO**	**α (eV)**	**β (V)**	**γ**	**MIN**	**MAX**	**MAE**	**STD**
CH_3	-0.2931	-0.4701	0.5396	-0.1029	0.1932	0.0728	0.0908
CH_2	-0.3015	-0.3291	0.7309	-0.1585	0.2088	0.0805	0.1028
CH	-0.3458	-0.2939	0.9395	-0.2363	0.1750	0.1024	0.1278
C	-0.4042	-0.4739	1.2683	-0.1924	0.2641	0.1124	0.1420
NH_3	0.1188	0.4621	0.2368	-0.1097	0.0856	0.0509	0.0645
NH_2	-0.0441	0.5159	0.3993	-0.1105	0.1113	0.0452	0.0594
NH	-0.1988	0.5974	0.6447	-0.2666	0.1937	0.0937	0.1259
N	-0.3560	0.2356	1.0926	-0.2622	0.2124	0.1181	0.1472
H_2O	0.1066	0.4269	0.0741	-0.0919	0.0648	0.0426	0.0504
OH	0.0327	1.0864	0.2384	-0.1107	0.1338	0.0525	0.0669
O	-0.2075	0.8292	0.6256	-0.2927	0.1902	0.1021	0.1365
H	-0.0777	-0.0394	0.3275	-0.1715	0.0943	0.0559	0.0755
CO	0	0	1	–	–	–	–
CH_3OH	0.0946	0.4236	0.0935	-0.0985	0.0702	0.0425	0.0515
all but CO	–	–	–	*-0.2927*	*0.2641*	*0.0747*	*0.0977*
**k = C**	**α (eV)**	**β (V)**	**γ**	**MIN**	**MAX**	**MAE**	**STD**
CH_3	-0.0999	-0.2579	0.4083	-0.2106	0.1693	0.0747	0.1009
CH_2	-0.0644	-0.0539	0.5730	-0.1195	0.1765	0.0494	0.0732
CH	-0.0529	0.0539	0.7460	-0.1115	0.0594	0.0338	0.0470
C	0	0	1	–	–	–	–
NH_3	0.2196	0.5632	0.1662	-0.1353	0.0904	0.0636	0.0792
NH_2	0.0906	0.6688	0.3089	-0.0641	0.0883	0.0455	0.0547
NH	-0.0035	0.8332	0.5166	-0.1783	0.1159	0.0686	0.0857
N	-0.0148	0.6403	0.8672	-0.1172	0.0595	0.0434	0.0535
H_2O	0.1370	0.4579	0.0530	-0.0953	0.0621	0.0430	0.0520
OH	0.1089	1.1756	0.1878	-0.0798	0.1177	0.0510	0.0615
O	-0.0253	1.0544	0.5071	-0.2037	0.1167	0.0693	0.0911
H	0.0515	0.0954	0.2381	-0.2374	0.0797	0.0652	0.0908
CO	0.3691	0.3988	0.7478	-0.2036	0.1665	0.0848	0.1090
CH_3OH	0.1318	0.4622	0.0677	-0.1026	0.0674	0.0429	0.0536
all but C	–	–	–	*-0.2374*	*0.1765*	*0.0565*	*0.073*

References.

(1) Kresse, G.; Hafner, J. Ab Initio Molecular Dynamics for Liquid Metals. *Phys. Rev. B* **1993**, *47*, 558–561. https://doi.org/10.1103/PhysRevB.47.558.

(2) Kresse, G.; Furthmüller, J. Efficiency of Ab-Initio Total Energy Calculations for Metals and Semiconductors Using a Plane-Wave Basis Set. *Comput. Mater. Sci.* **1996**, *6*, 15–50. https://doi.org/10.1016/0927-0256(96)00008-0.

(3) Kresse, G.; Furthmüller, J. Efficient Iterative Schemes for Ab Initio Total-Energy Calculations Using a Plane-Wave Basis Set. *Phys. Rev. B* **1996**, *54*, 11169–11186. https://doi.org/10.1103/PhysRevB.54.11169.

(4) Dion, M.; Rydberg, H.; Schröder, E.; Langreth, D. C.; Lundqvist, B. I. Van Der Waals Density Functional for General Geometries. *Phys. Rev. Lett.* **2004**, *92*, 246401. https://doi.org/10.1103/PhysRevLett.92.246401.

(5) Klimeš, J.; Bowler, D. R.; Michaelides, A. Chemical Accuracy for the van Der Waals Density Functional. *J. Phys. Condens. Matter* **2010**, *22*, 022201. https://doi.org/10.1088/0953-8984/22/2/022201.

(6) Klimeš, J.; Bowler, D. R.; Michaelides, A. Van Der Waals Density Functionals Applied to Solids. *Phys. Rev. B - Condens. Matter Mater. Phys.* **2011**, *83* (19), 1–13. https://doi.org/10.1103/PhysRevB.83.195131.

(7) Blöchl, P. Projector Augmented-Wave Method. **1994**, *50*, 17953.

(8) Kresse, G.; Joubert, D. From Ultrasoft Pseudopotentials to the Projector Augmented-Wave Method. *Phys. Rev. B* **1999**, *59*, 1758–1775. https://doi.org/10.1103/PhysRevB.59.1758.

(9) Darby, M. T.; Réocreux, R.; Sykes, E. C. H.; Michaelides, A.; Stamatakis, M. Elucidating the Stability and Reactivity of Surface Intermediates on Single-Atom Alloy Catalysts. *ACS Catal.* **2018**, *8* (6), 5038–5050. https://doi.org/10.1021/acscatal.8b00881.

(10) Monkhorst, H. J.; Pack, J. D. Special Points for Brillouin-Zone Integrations. *Phys. Rev. B* **1976**, *13*, 5188–5192. https://doi.org/10.1103/PhysRevB.13.5188.

(11) Perdew, J. P.; Burke, K.; Ernzerhof, M. Generalized Gradient Approximation Made Simple. *Phys. Rev. Lett.* **1996**, *77* (18), 3865–3868. https://doi.org/10.1103/PhysRevLett.77.3865.

(12) Sanville, E.; Kenny, S. D.; Smith, R.; Henkelman, G. Improved Grid-Based Algorithm for Bader Charge Allocation. *J. Comput. Chem.* **2007**, *28* (5), 899–908. https://doi.org/10.1002/jcc.20575.

(13) Tang, W.; Sanville, E.; Henkelman, G. A Grid-Based Bader Analysis Algorithm without Lattice Bias. *J. Phys. Condens. Matter* **2009**, *21* (8), 084204. https://doi.org/10.1088/0953-8984/21/8/084204.

(14) Henkelman, G.; Arnaldsson, A.; Jónsson, H. A Fast and Robust Algorithm for Bader Decomposition of Charge Density. *Comput. Mater. Sci.* **2006**, *36* (3), 354–360. https://doi.org/10.1016/j.commatsci.2005.04.010.

(15) Yu, M.; Trinkle, D. R. Accurate and Efficient Algorithm for Bader Charge Integration. *J. Chem. Phys.* **2011**, *134* (6), 064111. https://doi.org/10.1063/1.3553716.

(16) Hirshfeld, F. L. Bonded-Atom Fragments for Describing Molecular Charge Densities. *Theor. Chim. Acta* **1977**, *44* (2), 129–138. https://doi.org/10.1007/BF00549096.

(17) Manz, T. A.; Limas, N. G. Introducing DDEC6 Atomic Population Analysis: Part 1. Charge Partitioning Theory and Methodology. *RSC Adv.* **2016**, *6* (53), 47771–47801. https://doi.org/10.1039/C6RA04656H.

(18) Limas, N. G.; Manz, T. A. Introducing DDEC6 Atomic Population Analysis: Part 2. Computed Results for a Wide Range of Periodic and Nonperiodic Materials. *RSC Adv.* **2016**, *6* (51),

45727–45747. https://doi.org/10.1039/C6RA05507A.
(19) Steinmann, S. N.; Corminboeuf, C. A Generalized-Gradient Approximation Exchange Hole Model for Dispersion Coefficients. *J. Chem. Phys.* **2011**, *134* (4), 044117. https://doi.org/10.1063/1.3545985.
(20) Steinmann, S. N.; Corminboeuf, C. Comprehensive Benchmarking of a Density-Dependent Dispersion Correction. *J. Chem. Theory Comput.* **2011**, *7* (11), 3567–3577. https://doi.org/10.1021/ct200602x.
(21) Huheey, J. E.; Keiter, E. A.; Keiter, R. L. *Inorganic Chemistry : Principles of Structure and Reactivity*; 1993.
