# Supplementary material for: Stick or Spill? Scaling Relationships for the Binding Energies of Adsorbates on Single-Atom Alloy Catalysts
Source: J Phys Chem Lett. 2022 Aug 2;13(31):7314–9. doi: 10.1021/acs.jpclett.2c01519 (PMC9376958; doi:10.1021/acs.jpclett.2c01519)
Supplement: Supplementary file 2 — jz2c01519_si_002.pdf [file jz2c01519_si_002.pdf]

jz-2022-01519y.R1

Name: Peer Review Information for "Stick or Spill? Scaling Relationships for the Binding Energies of Adsorbates on Single-Atom Alloy Catalysts"

First Round of Reviewer Comments

Reviewer: 1

Comments to the Author

This paper discusses the development of a deeper understanding of the factors contributing to spillover between guest and host sites in single-atom alloy (SAA) catalysts. An analysis of spillover energy (SOE, difference in adsorption energy between the two sites) is presented for a variety of SAAs. It is found that combining electrostatic and covalent descriptors of the interaction allows for an intuitive and reasonably accurate prediction of SOE. I think this could be a good fit for a Phys Chem Letter, teaching readers how to think about SOEs with a succinct demonstration. That said I would recommend a few edits to strengthen the arguments and conclusions:

1) Spillover is often described in other work on SAAs (much by the current authors) as entropy-driven. Can they add some commentary about the typical magnitudes of configurational entropy gain with spillover to allow for assessment of how large an SOE would be needed to keep an adsorbate on the dopant site for some typical conditions?

2) The conclusions are somewhat limited by only including the three d9 and a single d8 dopants. I think the analysis could carry more weight if expanded to a few more elements.

3) Some of the original papers leading to d-band theory go through extended derivation arguing that differences in adsorption energy can be broken into contributions from (i) differences in the one-electron energy levels, and (ii) differences in electrostatic interaction (which are neglected or approximated constant comparing across pure metals etc). So I would say the finding that adding the electrostatic contribution improves accuracy is quite expected (not to diminish the nice insights of this paper). It would probably be a good idea to mention and cite for example Phys Rev B 35, 7423, 1987 and Rep. Prog Phys, 53, 1253, 1990.

4) I was curious to what extent the dopant atoms retain similar charges after adsorption. If adsorbates (particularly O or OH being the most electronegative) steal charge away, can this be a significant contributor to scatter in the data and should it be addressed in attributing the “character” of the species as covalent vs electrostatic as in Fig 3?

5) I am less familiar with the DDEC6 and HD charge allocation schemes, but can the authors comment as to why these correlate less well than Bader? Should this be a cause for concern?

Reviewer: 2

Comments to the Author

This paper focuses on an interesting question of the spillover effect of adsorbates on single atom alloys. The SAA is an important, and emerging catalysts for selectively heterogeneous reactions. Authors found that the bader charge on the dopant metal and the carbon binding energy are two important factors that can accurately capture the defined spillover energy. The analysis is in good details. However, there are a few concerns particularly the novelty of the approach.

1. Both Bader charge and *C binding energy are not proper type of features that can be used in practical design. Other physical properties and coordination have been widely used in catalysis often in the context of machine learning models with large data.

2. The discussion of electrostatic and covalent contributions to binding at single atoms is an extension of previous work. Thus, the novelty of mechanism and feature choices is rather limited.

3. One important thing about the two factor regression is the possible correlation of two features, i.e. bader charge and *C binding. It seems authors used bader charge as the first important feature and use *C for residual fitting. Such approach is arbitrary and might be biased.

4. It claimed that the charge inclusion scaling is more flexible than traditional scaling because the traditional scaling only correlates binding energy of related fragments. This is slightly not true because scaling of unrelated fragments is also common. From this perspective, the charge inclusion scaling does not provide new capability or insights.

Reviewer: 3

Comments to the Author

In this manuscript, the authors report two simple descriptors for the prediction of adsorbate spillover energy barriers from dopant to host sites on single atom alloys. The adsorption energies were calculated using DFT for 14 common adsorbates on a host of a coinage metal (Cu, Ag, and Au) doped with a single atom of Ni, Pd, Pt or Rh. The results showed that the Bader charges of the dopant metal and the spillover energy of carbon on the surface were effective descriptors for the spillover energy of the specific adsorbent. The results presented are of scientific significance as they could aid in the rational design of single atom alloys for catalytic applications. Moreover, the manuscript provides insight into the importance of the charges of the dopant atom on the stability of the adsorbed intermediates. Generally, the manuscript is well-written and in excellent scholarly form. The proposed methodology is clearly explained, and shows a simple scheme for catalyst screening, thus I believe it merits publication in The Journal of Physical Chemistry Letters.

Suggestions, comments, and questions:

1. The equation in Figure 1d is missing the x.

2. Figures 1b and 1c provide the same information, the inclusion of both Figures seems redundant.

3. Even though it is widely available information, please include the source for the data in Table S2.

4. Why were the values of H, C and N electronegativities included in Table S2. These values were not mentioned/used in the manuscript.

5. It is mentioned that the "The SOE is defined as the energy required as the energy required for an adsorbate to migrate from the dopant to a site on the host metal", it should be stated that both sites correspond to the most favorable adsorption sites.

6. Why was optB86b compared to PBE to estimate the vdW contribution to the energy change instead of comparing it to B86b?

7. In line 46 of page 4 it says that for the sample values the slope for the electrostatic contribution should be around 1V, however it would be closer to 0.7V with the assumptions made.

8. In the case of the electrostatic contribution, it is shown that it does not depend solely on the charge of the adsorbent but also on the charge of the adsorbate. Why doesn't this adsorbent charge have an effect on the descriptors?

9. Also, why only the charge of the dopant atom is considered since equation (ii) is a change in energy and equation (iii) is not

10. Why is the partitioning scheme used to calculate the charges so important for the quality of the correlation?

11. Would the conclusions of this work change if the catalyst were in liquid phase? There should be at least some comments on that.

12. The analysis presented in the manuscript focuses mainly on the coefficient of determination. However, it has been shown that overreliance on this parameter can lead to selection bias. The mean average error or other performance metrics should also be mentioned when analyzing the results. For instance, in Figure 2 it seems that in some cases high values of R2 are obtained but the dispersion is at least as large as those of the CHx adsorbates.

13. It would be useful if different symbols were used for the different host materials when presenting the results. Also, it might be useful to identify in Figure 2 (and Figures S2-S4) which systems correspond to the outliers of the overall trend.

14. The equation in Figure S4 for C should be written as y = x

15. What would be the difference in terms of the MAE of performing a multilinear regression with both descriptors instead of using the two-step regression approach proposed.

Author's Response to Peer Review Comments:

London, July 19th 2022

Revision of manuscript jz-2022-01519y

Dear Prof. Editor,

We thank you and the reviewers for your time and detailed review of our paper titled “Stick or Spill? Scaling Relationships for the Binding Energies of Adsorbates on Single-Atom Alloy Catalysts”. We addressed all the points raised by the reviewers and have attached to this letter a point-by-point response to each comment. The changes to the manuscript are highlighted in yellow.

We believe that this improved version of our manuscript would be of broad interest to the readership of *The Journal of Physical Chemistry Letters* and would motivate further research in the field.

Thank you for the consideration.
Sincerely yours,

Dr Romain Réocreux
Thomas Young Centre and Department of Chemical Engineering, University College London, UK
Yusuf Hamied Department of Chemistry, University of Cambridge, Cambridge, UK.

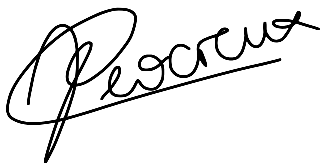

Editorial revision:

1) Supporting Information Statement: A brief, nonsentence description of the actual contents of each supporting information file is required. This description should be labeled Supporting Information and should appear before the Acknowledgement and Reference sections. Examples of sufficient and insufficient descriptions are as follows:
*Examples of sufficient descriptions: "Supporting Information: 1H NMR spectra for all compounds" or "Additional experimental details, materials, and methods, including photographs of experimental setup".
*Examples of insufficient descriptions: "Supporting Information: Figures S1-S3" or "Additional figures as mentioned in the text".

We have added the following to the manuscript:

Supporting Information
Computational details, tables including charges (Bader, HD and DDEC6) and SOEs, parameters and accuracy of the fitted model.

2) Title: Title must match in three places: (1) manuscript file, (2) supporting information, and (3) ACS Paragon Plus.

This has been fixed.

3) References: In both the main file and the supporting information, fix the style of all references to use JPCL formatting (check all references carefully). ***JPC Letters reference formatting requires that journal references should contain: () around numbers, author names, article title (titles entirely in title case or entirely in lower case), abbreviated journal title (italicized), year (bolded), volume (italicized), and pages (first-last). Book references should contain author names, book title (in the same pattern), publisher, city, and year.

We have checked the references.

4) Supporting Information: Please number SI pages in the following format: "S1, S2…"

We have added page numbering to the SI.

Reviewer(s)' Comments to Author:

Reviewer: 1

Recommendation: This paper is probably publishable, but major revision is needed; I do not need to see future revisions.

Comments:
This paper discusses the development of a deeper understanding of the factors contributing to spillover between guest and host sites in single-atom alloy (SAA) catalysts. An analysis of spillover energy (SOE, difference in adsorption energy between the two sites) is presented for a variety of SAAs. It is found that combining electrostatic and covalent descriptors of the interaction allows for an intuitive and reasonably accurate prediction of SOE. I think this could be a good fit for a Phys Chem Letter, teaching readers how to think about SOEs with a succinct demonstration. That said I would recommend a few edits to strengthen the arguments and conclusions:

We thank the reviewer for their positive feedback. We hope the following responses address their comments and our edits strengthen our manuscript.

1) Spillover is often described in other work on SAAs (much by the current authors) as entropy-driven. Can they add some commentary about the typical magnitudes of configurational entropy gain with spillover to allow for assessment of how large an SOE would be needed to keep an adsorbate on the dopant site for some typical conditions?

Although entropy always facilitates spillover, it is not usually the main driving force because the entropic contribution is very limited compared to the range of SOE determined for the considered 12 Single-Atom Alloy surfaces.

We have added the following section to the SI (referred to in the introduction).

Considering top sites only, the spillover entropy (from dopant, with surface loading δ, to host sites) can be approximated by the following formula:

$$\Delta_{SO}S = -k_B \ln\left(\frac{\delta}{1-\delta}\right)$$

For typical dopant loadings of $\delta = 5\%$ to $\delta = 0.1\%$, the spillover entropy can range from 24 to 57 J·mol^{-1}·K^{-1}. At room temperature, this translates into a $T\Delta_{SO}S$ contribution to the spillover free energy that ranges from 0.07 eV to 0.18 eV. This contribution only plays a role for small SOEs and becomes more significant at higher temperatures or for very low dopant concentrations (unlike the SOE). For instance, the SOEs of C range from 0.12 eV to 2.41 eV.

2) The conclusions are somewhat limited by only including the three d9 and a single d8 dopants. I think the analysis could carry more weight if expanded to a few more elements.

We have only focused on the dopants that have been extensively studied experimentally. Now that we have disentangled the electrostatic and covalent contributions to the SOE, we are in the process of extending our results to all the dopants of the d-block. We are currently developing a model aiming at describing the covalent contribution using electronic descriptors related to the intrinsic electronic properties of SAAs rather than their carbophilicity. This is beyond the scope of the current letter.

3) Some of the original papers leading to d-band theory go through extended derivation arguing that differences in adsorption energy can be broken into contributions from (i) differences in the one-electron energy levels, and (ii) differences in electrostatic interaction (which are neglected or approximated constant comparing across pure metals etc). So I would say the finding that adding the electrostatic contribution improves accuracy is quite expected (not to diminish the nice insights of this paper). It would probably be a good idea to mention and cite for example Phys Rev B 35, 7423, 1987 and Rep. Prog Phys, 53, 1253, 1990.

We thank the reviewer for suggesting these two papers.

It is important to note that transition metals do not exhibit atomic charges at their surface **in its pristine form (i.e. in the absence of adsorbed species)**. On SAAs however, some dopants can exhibit large atomic charges, making the inclusion of the electrostatic term crucial for the understanding of the relative stability of species between host and dopant sites.

The first paper suggested by the reviewer describes the development of a density functional model for the electronic properties of bulk metals and surfaces (phonon, surface energies, cohesive energies, …). It does not elaborate on the interaction of adsorbates on metal surfaces. We have therefore decided not to include it in our manuscript. We have however cited the second paper which is very relevant to our work (ref 23).

4) I was curious to what extent the dopant atoms retain similar charges after adsorption. If adsorbates (particularly O or OH being the most electronegative) steal charge away, can this be a significant contributor to scatter in the data and should it be addressed in attributing the "character" of the species as covalent vs electrostatic as in Fig 3?

We have performed two extra Bader charge calculations for O on NiAu (with initial charge of 0.32e) and PtCu (initial charge of -0.61e). When O is adsorbed on the threefold dopant-host mixed site, the three atoms involved in the adsorption site give electrons to O, resulting in dopant charges being shifted to more positive values (0.52e for NiAu and -0.29 for PtCu). This reflects a more complex bonding mechanism involving a significant redistribution of the electronic density and could indeed explain the scatter observed in the plots and the significance of the covalent term for this adsorbate.

We have added a sentence to mention the effect of adsorption of O on NiAu and PtCu SAAs:

This is, of course, an oversimplified scenario, as the electronic structure of the adsorbate is not frozen and is affected by interactions with the surface. For instance, further Bader charge calculations on adsorbed states show that O takes 0.20e from Ni on NiAu and 0.32e from Pt on PtCu. Nevertheless, if the electrostatic term dominates, the dopant charge is likely to remain a good descriptor and the SOE should correlate with the dopant charge as predicted by Eq. (iii).

5) I am less familiar with the DDEC6 and HD charge allocation schemes, but can the authors comment as to why these correlate less well than Bader? Should this be a cause for concern?

There is no definite definition for atomic charges. The reason for that is that atomic charges are not observables and are therefore not associated with a well-defined operator in quantum physics. Definitions of charges vary greatly depending on applications. For example, the simplest model that assigns all the electrons of the covalent bond to the most electronegative atom gives atomic charges that are commonly referred to as oxidation states. These are useful to sort, qualitatively, species based on their electrochemical properties. Another simple approach, that essentially works for molecules, is to assign point charges (thereby reducing the complexity of the spatial extension of the electronic density) to reproduce electric dipoles. However, these methods ignore the complexity of chemical bonding and how it affects the electronic density locally.

The methods we have considered in the paper are based on the electronic density. The HD method compares the spherically averaged electron density around each atom of a system with gas phase atoms. Since the size of the electronic density affects the dispersion interaction between molecules, this scheme is used in the dDsC correction for GGA functionals. Charges can also be derived using this scheme.

The DDEC6 method follows a similar approach (although comparing to reference ions) while imposing the point charge to approximate the electrostatic potential created by each atom. We have tried this scheme as we have been analyzing electrostatic interactions.

Yet, when the electronic density is delocalized and flat between pairs of atoms such as in metals ("sea" of electrons), spherical averaging may be arbitrary. The heterogenous catalysis community working on metal surfaces most often adopt the Bader Charge as a descriptor for interactions or spectroscopic features. This scheme defines the border between two atoms as the surface of null gradient of the electron density and integrates the density over the resulting volume. There is no assumption regarding the shape of atoms, or the symmetry of the averaging of the electron density. It would be more concerning if the Bader charges were not a good descriptor for electrostatic effects. Unfortunately, the other partitioning schemes that we have tried do not perform well for our charge-inclusive linear scaling relationships.

Additional Questions:
Urgency: High

Significance: High

Novelty: High

Scholarly Presentation: High

Is the paper likely to interest a substantial number of physical chemists, not just specialists working in the authors' area of research?: Yes

Reviewer: 2

Recommendation: Reconsider as an article in The Journal of Physical Chemistry A/B/C.

Comments:
This paper focuses on an interesting question of the spillover effect of adsorbates on single atom alloys. The SAA is an important, and emerging catalysts for selectively heterogeneous reactions. Authors found that the bader charge on the dopant metal and the carbon binding energy are two important factors that can accurately capture the defined spillover energy. The analysis is in good details. However, there are a few concerns particularly the novelty of the approach.

We thank the reviewer for their feedback. We have clarified certain aspects of the manuscript to address their concerns.

1. Both Bader charge and *C binding energy are not proper type of features that can be used in practical design. Other physical properties and coordination have been widely used in catalysis often in the context of machine learning models with large data.

It is true that other features have been widely used in catalysis. They include the d-band centre or the generalised coordination number. There is however a long tradition, dating back to Prof. Sabatier (first half of 20th century), and a significant body of literature using binding energies of identified adsorbates to predict catalytic performance (represented as "activity *vs* binding energy" volcano plots). However, the complexity of catalytic systems usually requires consideration of the binding energies of more than one adsorbate. The work by Nørskov and co-workers has shown, among others, that 2D volcano plots, using the binding energies of two adsorbates, capture catalytic performance more accurately. In our letter, we identify the Bader charge as an alternative descriptor for the binding of species on SAAs, which can advantageously easily be computed on a clean surface model. This approach enables us to disentangle the purely covalent contribution (the electronegativities of carbon and late transition metals being similar) from the electrostatic contribution in the binding mechanism of a range of adsorbates. Finally, the values of these descriptors could easily be used, like the electronegativity of an atom or its van der Waals radius, as input parameters for machine learning models.

We have revised the paper to highlight the role of binding energies in catalyst design:

The success of these models results from their simplicity: only a few parameters (e.g., the d-band centre or the binding energies of C and O) are needed to provide semi-quantitative predictions regarding the performance of catalysts.[16,26–28]

Ref. 16 is an example where the binding energy of O and C (determined using machine learning trained on DFT data) can be used to predict reactivity trends for ethanol dehydrogenation.
Ref 28 shows how going from Pd to the more oxophilic Fe (larger binding energy of O) makes the C-O cleavage of aromatic oxygenates easier.

2. The discussion of electrostatic and covalent contributions to binding at single atoms is an extension of previous work. Thus, the novelty of mechanism and feature choices is rather limited.

The theoretical evidence of surface charges, the use of such charges to predict the stability of adsorbates, and the universality of our approach to any adsorbate contributes to the novelty of our work, which will be of great interest for theoreticians and experimentalists working in the growing field of SAA catalysis.

More specifically, we agree with the reviewer that describing the binding of two entities as a combination of electrostatic and covalent contributions is very common in homogeneous catalysis and some areas of heterogeneous catalysis. On metal surfaces, however, models usually assume that the electronic structure is rather homogeneous. Simple adsorption models on metal surfaces describe the interaction of adsorbates modelling the sp band as a jellium and switching on the interaction with the d-band with perturbation theory (see ref 23, suggested by reviewer #1). These early models still play an important role in our understanding of chemical bonds on metal surfaces, even today. On SAAs we show, via the Bader charge analysis, that dopants exhibit significant charges. Previous experimental work also stated, prior to our study, that some degree of charge redistribution must occur on SAAs to explain spectroscopic features (e.g., ref 39). Our work provides theoretical evidence for dopant charges. More importantly it shows that we can completely describe the adsorption energy of any adsorbate (whether bound to the surface via H, C, N, or O) using the binding energy of carbon and the dopant charge, thereby disentangling the covalent and electrostatic contributions to the binding. Two-parameter models already exist, they typically use the binding energies of two species (which might partly be correlated) relevant to specific applications (N and H for nitrogen reduction; C and O for CO_2 reduction) but only provide limited insight into the nature of the binding mechanism.

We have rephrased the conclusion to highlight the novelty of our work:

In conclusion, through a detailed study of common adsorbates on SAA surfaces, we have furthered understanding on the relative binding of adsorbates to host and dopant sites. We have shown that the electronegativity difference between the dopant and the host atoms results in the formation of atomic charges localised on the dopant atom, with the countercharge fully delocalised on the host. Our work therefore provides theoretical rationalisation of experimental observations suggesting the presence of charges located at the dopant of SAA surfaces. […] The combination of these two descriptors captures, ~~in an intuitive way~~ by disentangling the covalent and electrostatic contributions, the main bonding mechanisms of surface species on SAAs, regardless of whether the species are chemically related to C or not. In that sense, our charge-inclusive Thermo-Chemical Scaling relationships offer more versatility and transversality on SAAs than traditional Thermo-Chemical Scaling relationships, which tend to solely correlate the binding energies of chemically related fragments.[31,49]

3. One important thing about the two factor regression is the possible correlation of two features, i.e. bader charge and *C binding. It seems authors used bader charge as the first important feature and use *C for residual fitting. Such approach is arbitrary and might be biased.

There is no correlation between the Bader Charges (R^2=0.06) and the SOE of C (see last panel of Figure 2), hence our choice for the SOE of C as a second parameter.

We agree with the referee that using the Bader charges as the first important descriptor can be biased, especially for species with a dominant covalent character. We have therefore re-analysed the data considering a multilinear regression approach using the identified descriptors. The multilinear regression results in even better agreement between DFT computed SOEs and fitted SOEs.

We have corrected the main text accordingly:

To build a quantitative surrogate model that simultaneously captures the effects of both bonding mechanisms, we have performed a multilinear regression using both the dopant charge and the SOE of C as descriptors ~~we have adopted a two-step regression approach. Starting from the linear regressions of the SOEs as a function of the dopant charge (Figure 2), we have regressed the residuals on the SOE of C.~~ The parameters of the regression~~s~~ are given in Table S5. Figure 3d shows the parity plot of the fitted SOEs against the DFT-computed SOEs. The SOEs are reproduced with a mean absolute error (MAE) of ~~0.08~~ 0.06 eV and a standard deviation of ~~0.11~~ 0.07 eV. […] We have therefore considered the SOEs of H and CO, both at the covalent end of the scale shown in Figure 3b, as alternatives for the covalent parameter of the surrogate model ~~(Table S6)~~. The SOE of H turns out to give a poor description of the non-electrostatic contribution (Table S~~6~~5). The SOE of CO, albeit not as good as the SOE of C, performs ~~relatively better~~ well with a MAE of ~~0.12~~ 0.07 eV and a standard deviation of ~~0.17~~ 0.10 eV. Thus, the SOE of CO could also be used~~, at least qualitatively,~~ instead of the SOE of C, should the latter be unknown.

We have also added a full description of the parameters and accuracy of the new multilinear model in Table S5 (now replacing the previous Tables S5-S6).

Table S5. *Parameters of the multilinear regression* $SOE = \alpha + \beta \times q_d + \gamma \times SOE_k$ *with* $k \in \{H, CO, C\}$. *MIN, MAX, MAE and STD stand for the minimum and maximum deviations, the mean absolute error, and the standard deviation. MAE and STD are not reported for the adsorbate whose SOE is used as a descriptor.*

adsorbates	***Parameters and Accuracy of the surrogate model***						
k = H	***α (eV)***	***β (V)***	***γ***	***MIN***	***MAX***	***MAE***	***STD***
CH_3	*-0.1154*	*-0.3705*	*1.5045*	*-0.1425*	*0.1523*	*0.0621*	*0.0805*
CH_2	*-0.0097*	*-0.1583*	*1.8901*	*-0.2395*	*0.3432*	*0.1108*	*0.1581*
CH	*0.0702*	*-0.0457*	*2.3113*	*-0.3499*	*0.5354*	*0.1687*	*0.2392*
C	*0.1784*	*-0.1243*	*3.0598*	*-0.4974*	*0.7574*	*0.2320*	*0.3256*
NH_3	*0.2453*	*0.5398*	*0.5200*	*-0.1620*	*0.0973*	*0.0787*	*0.0941*
NH_2	*0.1391*	*0.6258*	*0.9641*	*-0.1602*	*0.1895*	*0.0891*	*0.1096*
NH	*0.0840*	*0.7658*	*1.5939*	*-0.3439*	*0.3578*	*0.1345*	*0.1853*
N	*0.1427*	*0.5345*	*2.6453*	*-0.3976*	*0.6393*	*0.2139*	*0.2896*
H_2O	*0.1719*	*0.4692*	*0.0887*	*-0.1026*	*0.0631*	*0.0499*	*0.0597*
OH	*0.1598*	*1.1645*	*0.5242*	*-0.1444*	*0.1868*	*0.0703*	*0.0959*
O	*0.0806*	*1.0022*	*1.5072*	*-0.3708*	*0.3799*	*0.1448*	*0.1992*
H	*0*	*0*	*1*	–	–	–	–
CO	*0.4356*	*0.2591*	*2.4811*	*-0.2464*	*0.4033*	*0.1747*	*0.2078*
CH_3OH	*0.1685*	*0.4711*	*0.1360*	*-0.1137*	*0.0696*	*0.0528*	*0.0641*
all but H	–	–	–	*-0.4974*	*0.7574*	*0.1217*	*0.1754*
k = CO	***α (eV)***	***β (V)***	***γ***	***MIN***	***MAX***	***MAE***	***STD***
CH_3	*-0.2931*	*-0.4701*	*0.5396*	*-0.1029*	*0.1932*	*0.0728*	*0.0908*
CH_2	*-0.3015*	*-0.3291*	*0.7309*	*-0.1585*	*0.2088*	*0.0805*	*0.1028*
CH	*-0.3458*	*-0.2939*	*0.9395*	*-0.2363*	*0.1750*	*0.1024*	*0.1278*
C	*-0.4042*	*-0.4739*	*1.2683*	*-0.1924*	*0.2641*	*0.1124*	*0.1420*
NH_3	*0.1188*	*0.4621*	*0.2368*	*-0.1097*	*0.0856*	*0.0509*	*0.0645*
NH_2	*-0.0441*	*0.5159*	*0.3993*	*-0.1105*	*0.1113*	*0.0452*	*0.0594*
NH	*-0.1988*	*0.5974*	*0.6447*	*-0.2666*	*0.1937*	*0.0937*	*0.1259*
N	*-0.3560*	*0.2356*	*1.0926*	*-0.2622*	*0.2124*	*0.1181*	*0.1472*
H_2O	*0.1066*	*0.4269*	*0.0741*	*-0.0919*	*0.0648*	*0.0426*	*0.0504*
OH	*0.0327*	*1.0864*	*0.2384*	*-0.1107*	*0.1338*	*0.0525*	*0.0669*
O	*-0.2075*	*0.8292*	*0.6256*	*-0.2927*	*0.1902*	*0.1021*	*0.1365*
H	*-0.0777*	*-0.0394*	*0.3275*	*-0.1715*	*0.0943*	*0.0559*	*0.0755*
CO	*0*	*0*	*1*	–	–	–	–
CH_3OH	*0.0946*	*0.4236*	*0.0935*	*-0.0985*	*0.0702*	*0.0425*	*0.0515*
all but CO	–	–	–	*-0.2927*	*0.2641*	*0.0747*	*0.0977*

k = C	*α (eV)*	*β (V)*	*γ*	*MIN*	*MAX*	*MAE*	*STD*
CH_3	*-0.0999*	*-0.2579*	*0.4083*	*-0.2106*	*0.1693*	*0.0747*	*0.1009*
CH_2	*-0.0644*	*-0.0539*	*0.5730*	*-0.1195*	*0.1765*	*0.0494*	*0.0732*
CH	*-0.0529*	*0.0539*	*0.7460*	*-0.1115*	*0.0594*	*0.0338*	*0.0470*
C	*0*	*0*	*1*	*–*	*–*	*–*	*–*
NH_3	*0.2196*	*0.5632*	*0.1662*	*-0.1353*	*0.0904*	*0.0636*	*0.0792*
NH_2	*0.0906*	*0.6688*	*0.3089*	*-0.0641*	*0.0883*	*0.0455*	*0.0547*
NH	*-0.0035*	*0.8332*	*0.5166*	*-0.1783*	*0.1159*	*0.0686*	*0.0857*
N	*-0.0148*	*0.6403*	*0.8672*	*-0.1172*	*0.0595*	*0.0434*	*0.0535*
H_2O	*0.1370*	*0.4579*	*0.0530*	*-0.0953*	*0.0621*	*0.0430*	*0.0520*
OH	*0.1089*	*1.1756*	*0.1878*	*-0.0798*	*0.1177*	*0.0510*	*0.0615*
O	*-0.0253*	*1.0544*	*0.5071*	*-0.2037*	*0.1167*	*0.0693*	*0.0911*
H	*0.0515*	*0.0954*	*0.2381*	*-0.2374*	*0.0797*	*0.0652*	*0.0908*
CO	*0.3691*	*0.3988*	*0.7478*	*-0.2036*	*0.1665*	*0.0848*	*0.1090*
CH_3OH	*0.1318*	*0.4622*	*0.0677*	*-0.1026*	*0.0674*	*0.0429*	*0.0536*
all but C	*–*	*–*	*–*	*-0.2374*	*0.1765*	*0.0565*	*0.073*

4. It claimed that the charge inclusion scaling is more flexible than traditional scaling because the traditional scaling only correlates binding energy of related fragments. This is slightly not true because scaling of unrelated fragments is also common. From this perspective, the charge inclusion scaling does not provide new capability or insights.

Although "accidental" linear scaling relationships exist between the binding energies of two non-chemically related adsorbates, this is not the rule. A recent article by Nørskov and co-workers identifies the limits of the linear scaling correlations for adsorbates bound via different atoms on pure metal surfaces. We have added this reference in the conclusion of our article (ref 48).

Additional Questions:
Urgency: High

Significance: Moderate

Novelty: Moderate

Scholarly Presentation: High

Is the paper likely to interest a substantial number of physical chemists, not just specialists working in the authors' area of research?: Yes

Reviewer: 3

Recommendation: This paper is probably publishable, but major revision is needed; I do not need to see future revisions.

Comments:
In this manuscript, the authors report two simple descriptors for the prediction of adsorbate spillover energy barriers from dopant to host sites on single atom alloys. The adsorption energies were calculated using DFT for 14 common adsorbates on a host of a coinage metal (Cu, Ag, and Au) doped with a single atom of Ni, Pd, Pt or Rh. The results showed that the Bader charges of the dopant metal and the spillover energy of carbon on the surface were effective descriptors for the spillover energy of the specific adsorbent. The results presented are of scientific significance as they could aid in the

rational design of single atom alloys for catalytic applications. Moreover, the manuscript provides insight into the importance of the charges of the dopant atom on the stability of the adsorbed intermediates. Generally, the manuscript is well-written and in excellent scholarly form. The proposed methodology is clearly explained, and shows a simple scheme for catalyst screening, thus I believe it merits publication in The Journal of Physical Chemistry Letters.

We thank the reviewer for their very positive feedback.

Suggestions, comments, and questions:
1. The equation in Figure 1d is missing the x.

We have corrected Figure 1d (see next comment).

2. Figures 1b and 1c provide the same information, the inclusion of both Figures seems redundant.

We agree with the reviewer. We have removed the value of the charges in the bar plot, which is given here to highlight the trends between hosts and dopants. The heat map still provides the numerical values of the charges.

The new Figure 1 can be found below:

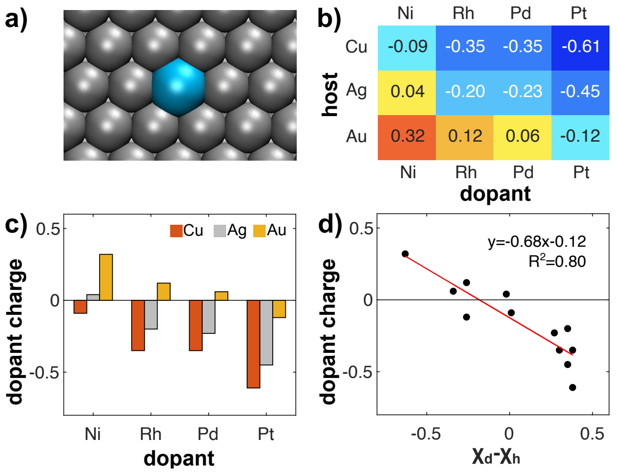

Figure 1. *Atomic charges (Bader analysis) in SAA surfaces. (a) Surface structure of the (111) facet of a SAA (dopant in cyan, host in grey). (b) Heatmap chart and (c) plot of the dopant charges (in units of e, the elementary charge) for Cu, Ag and Au based SAAs. (d) Correlation between the dopant charge and the electronegativity (χ) difference between the host (χ_h) and the dopant (χ_d) metals.*

3. Even though it is widely available information, please include the source for the data in Table S2.

We have added a textbook reference (ref 21 in the SI).

4. Why were the values of H, C and N electronegativities included in Table S2. These values were not mentioned/used in the manuscript.

These values were mentioned when discussing the classification with increasing electrostatic / covalent character (last sentence of last paragraph of page 5, just above Figure 3).

5. It is mentioned that the "The SOE is defined as the energy required as the energy required for an

adsorbate to migrate from the dopant to a site on the host metal", it should be stated that both sites correspond to the most favorable adsorption sites.

We thank the reviewer for spotting the lack of clarity of the definition of the SOE. We have corrected the text as follows:

The SOE is defined as the energy required for an adsorbate to migrate from the dopant atom site to ~~a~~ the most stable site on the host metal (Figure 2a)

6. Why was optB86b compared to PBE to estimate the vdW contribution to the energy change instead of comparing it to B86b?

This is an interesting question. "optB86b" on its own without the vdW component is simply the *exchange* functional for one particular non-local van der Waals density functional. It needs to be combined with an appropriate *correlation* functional so use on its own is not likely to yield accurate results. In this respect optB86b-vdW (and the other non-local van der Waals functionals in the same family) are distinct from the more empirical van der Waals correction schemes (like Grimme's D corrections) where a dispersion contribution is added to an established *exchange and correlation* functional. So, in the current study, to account for van der Waals interactions, we use the optB86b-vdW functional (which has a long, successful, and well validated track record for these types of systems) and for comparison purposes compare to another well-established functional, namely PBE, which does not account for van der Waals.

In the revised manuscript we have made the following changes:

~~Comparing the SOEs of OH computed with optB86b-vdW and PBE (the former providing an estimation of long-range dispersion interactions unlike the latter).~~ Comparing the SOEs of OH computed with two well-established functionals, namely PBE and optB86b-vdW,[40,45] is insightful. The correlation part of the latter functional is specifically designed to account for dispersion interactions, which are poorly described by the former. Despite this essential difference, the two sets of SOEs for OH are similar within 0.04 eV.

7. In line 46 of page 4 it says that for the sample values the slope for the electrostatic contribution should be around 1V, however it would be closer to 0.7V with the assumptions made.

We have changed 1 V to 0.7 V.

In this situation, one would expect from a purely electrostatic perspective, for $q_a \sim -0.1e$ and $d \sim 2$ Å, a linear variation of the SOE with respect to the dopant charge with a slope of about +~~1 V~~ 0.7 V.

8. In the case of the electrostatic contribution, it is shown that it does not depend solely on the charge of the adsorbent but also on the charge of the adsorbate. Why doesn't this adsorbent charge have an effect on the descriptors?

Adsorbate charges do influence the SOEs, hence the change of slope in our regressions.

9. Also, why only the charge of the dopant atom is considered since equation (ii) is a change in energy and equation (iii) is not

Equations (ii) and (iii) both refer to the SOE, which is, by definition, the difference between the binding energies of an adsorbate on two adsorption sites. Both equations correspond to energy changes. The electrostatic contribution on host sites in assumed to be zero as the host is not charged. We have clarified the derivation of the equation as follows:

$$SOE \simeq \Delta E_{cov} + \left(\frac{q_a}{4\pi\epsilon_0 d}\right) \times (0 - q_d) = \Delta E_{cov} - \left(\frac{q_a}{4\pi\epsilon_0 d}\right) \times q_d$$

We have also added the following sentence to the manuscript:

The electrostatic contribution on host sites in assumed to be zero as the host is not charged.

10. Why is the partitioning scheme used to calculate the charges so important for the quality of the correlation?

A similar question was raised by reviewer 1. Please see our detailed answer to comment 5 from reviewer 1.

11. Would the conclusions of this work change if the catalyst were in liquid phase? There should be at least some comments on that.

This is a very interesting question. Liquids can be modelled, as a first approximation, as dielectrics with a different permittivity. However, the spillover of adsorbates does not happen in the liquid phase, but at the interface between a liquid and a solid where the permittivity is not easy to evaluate without further (computationally expensive) simulations. Hydrogen bond networks in protic solvents might also play a significant role in the case of electron-rich adsorbates (bound to the surface via N or O). This is beyond the scope of our letter.

12. The analysis presented in the manuscript focuses mainly on the coefficient of determination. However, it has been shown that overreliance on this parameter can lead to selection bias. The mean average error or other performance metrics should also be mentioned when analyzing the results. For instance, in Figure 2 it seems that in some cases high values of R2 are obtained but the dispersion is at least as large as those of the CHx adsorbates.

Figures 2 and S2-4 only aim at identifying whether there are correlations between the SOEs and the Bader charges or the SOE of C. They do not aim at providing a quantitative model. The quantitative model is presented at the end of the article when considering the two contributions (electrostatic and covalent) at the same time. We have removed the R^2 values and added more statistical analysis results regarding the latter in Table S5 that replaces the previous Tables S5-S6

Table S5. *Parameters of the multilinear regression* $SOE = \alpha + \beta \times q_d + \gamma \times SOE_k$ *with* $k \in \{H, CO, C\}$. *MIN, MAX, MAE and STD stand for the minimum and maximum deviations, the mean absolute error, and the standard deviation. MAE and STD are not reported for the adsorbate whose SOE is used as a descriptor.*

adsorbates	***Parameters and Accuracy of the surrogate model***						
k = H	***α (eV)***	***β (V)***	***γ***	***MIN***	***MAX***	***MAE***	***STD***
CH_3	*-0.1154*	*-0.3705*	*1.5045*	*-0.1425*	*0.1523*	*0.0621*	*0.0805*
CH_2	*-0.0097*	*-0.1583*	*1.8901*	*-0.2395*	*0.3432*	*0.1108*	*0.1581*
CH	*0.0702*	*-0.0457*	*2.3113*	*-0.3499*	*0.5354*	*0.1687*	*0.2392*
C	*0.1784*	*-0.1243*	*3.0598*	*-0.4974*	*0.7574*	*0.2320*	*0.3256*
NH_3	*0.2453*	*0.5398*	*0.5200*	*-0.1620*	*0.0973*	*0.0787*	*0.0941*
NH_2	*0.1391*	*0.6258*	*0.9641*	*-0.1602*	*0.1895*	*0.0891*	*0.1096*
NH	*0.0840*	*0.7658*	*1.5939*	*-0.3439*	*0.3578*	*0.1345*	*0.1853*
N	*0.1427*	*0.5345*	*2.6453*	*-0.3976*	*0.6393*	*0.2139*	*0.2896*
H_2O	*0.1719*	*0.4692*	*0.0887*	*-0.1026*	*0.0631*	*0.0499*	*0.0597*
OH	*0.1598*	*1.1645*	*0.5242*	*-0.1444*	*0.1868*	*0.0703*	*0.0959*
O	*0.0806*	*1.0022*	*1.5072*	*-0.3708*	*0.3799*	*0.1448*	*0.1992*
H	*0*	*0*	*1*	*–*	*–*	*–*	*–*
CO	*0.4356*	*0.2591*	*2.4811*	*-0.2464*	*0.4033*	*0.1747*	*0.2078*
CH_3OH	*0.1685*	*0.4711*	*0.1360*	*-0.1137*	*0.0696*	*0.0528*	*0.0641*

all but H	–	–	–	-0.4974	0.7574	0.1217	0.1754
k = CO	***α (eV)***	***β (V)***	***γ***	***MIN***	***MAX***	***MAE***	***STD***
CH_3	-0.2931	-0.4701	0.5396	-0.1029	0.1932	0.0728	0.0908
CH_2	-0.3015	-0.3291	0.7309	-0.1585	0.2088	0.0805	0.1028
CH	-0.3458	-0.2939	0.9395	-0.2363	0.1750	0.1024	0.1278
C	-0.4042	-0.4739	1.2683	-0.1924	0.2641	0.1124	0.1420
NH_3	0.1188	0.4621	0.2368	-0.1097	0.0856	0.0509	0.0645
NH_2	-0.0441	0.5159	0.3993	-0.1105	0.1113	0.0452	0.0594
NH	-0.1988	0.5974	0.6447	-0.2666	0.1937	0.0937	0.1259
N	-0.3560	0.2356	1.0926	-0.2622	0.2124	0.1181	0.1472
H_2O	0.1066	0.4269	0.0741	-0.0919	0.0648	0.0426	0.0504
OH	0.0327	1.0864	0.2384	-0.1107	0.1338	0.0525	0.0669
O	-0.2075	0.8292	0.6256	-0.2927	0.1902	0.1021	0.1365
H	-0.0777	-0.0394	0.3275	-0.1715	0.0943	0.0559	0.0755
CO	0	0	1	–	–	–	–
CH_3OH	0.0946	0.4236	0.0935	-0.0985	0.0702	0.0425	0.0515
all but CO	–	–	–	-0.2927	0.2641	0.0747	0.0977
k = C	***α (eV)***	***β (V)***	***γ***	***MIN***	***MAX***	***MAE***	***STD***
CH_3	-0.0999	-0.2579	0.4083	-0.2106	0.1693	0.0747	0.1009
CH_2	-0.0644	-0.0539	0.5730	-0.1195	0.1765	0.0494	0.0732
CH	-0.0529	0.0539	0.7460	-0.1115	0.0594	0.0338	0.0470
C	0	0	1	–	–	–	–
NH_3	0.2196	0.5632	0.1662	-0.1353	0.0904	0.0636	0.0792
NH_2	0.0906	0.6688	0.3089	-0.0641	0.0883	0.0455	0.0547
NH	-0.0035	0.8332	0.5166	-0.1783	0.1159	0.0686	0.0857
N	-0.0148	0.6403	0.8672	-0.1172	0.0595	0.0434	0.0535
H_2O	0.1370	0.4579	0.0530	-0.0953	0.0621	0.0430	0.0520
OH	0.1089	1.1756	0.1878	-0.0798	0.1177	0.0510	0.0615
O	-0.0253	1.0544	0.5071	-0.2037	0.1167	0.0693	0.0911
H	0.0515	0.0954	0.2381	-0.2374	0.0797	0.0652	0.0908
CO	0.3691	0.3988	0.7478	-0.2036	0.1665	0.0848	0.1090
CH_3OH	0.1318	0.4622	0.0677	-0.1026	0.0674	0.0429	0.0536
all but C	–	–	–	-0.2374	0.1765	0.0565	0.073

13. It would be useful if different symbols were used for the different host materials when presenting the results. Also, it might be useful to identify in Figure 2 (and Figures S2-S4) which systems correspond to the outliers of the overall trend.

We have added a version of Figure 2 in the SI (Figure S2 – see below) that uses a colour code to identify the host of each data point. We have also edited Figures S4 (now Figure S5) in a similar way. We have kept the original Figure 2 in the main text as we think it is more important to analyse the data in terms of the degree of unsaturation of the adsorbates (which increases from left to right) and the nature of the atom bound to the surface (O for the red plots, N for the blue plots and C or H for the grey plots).

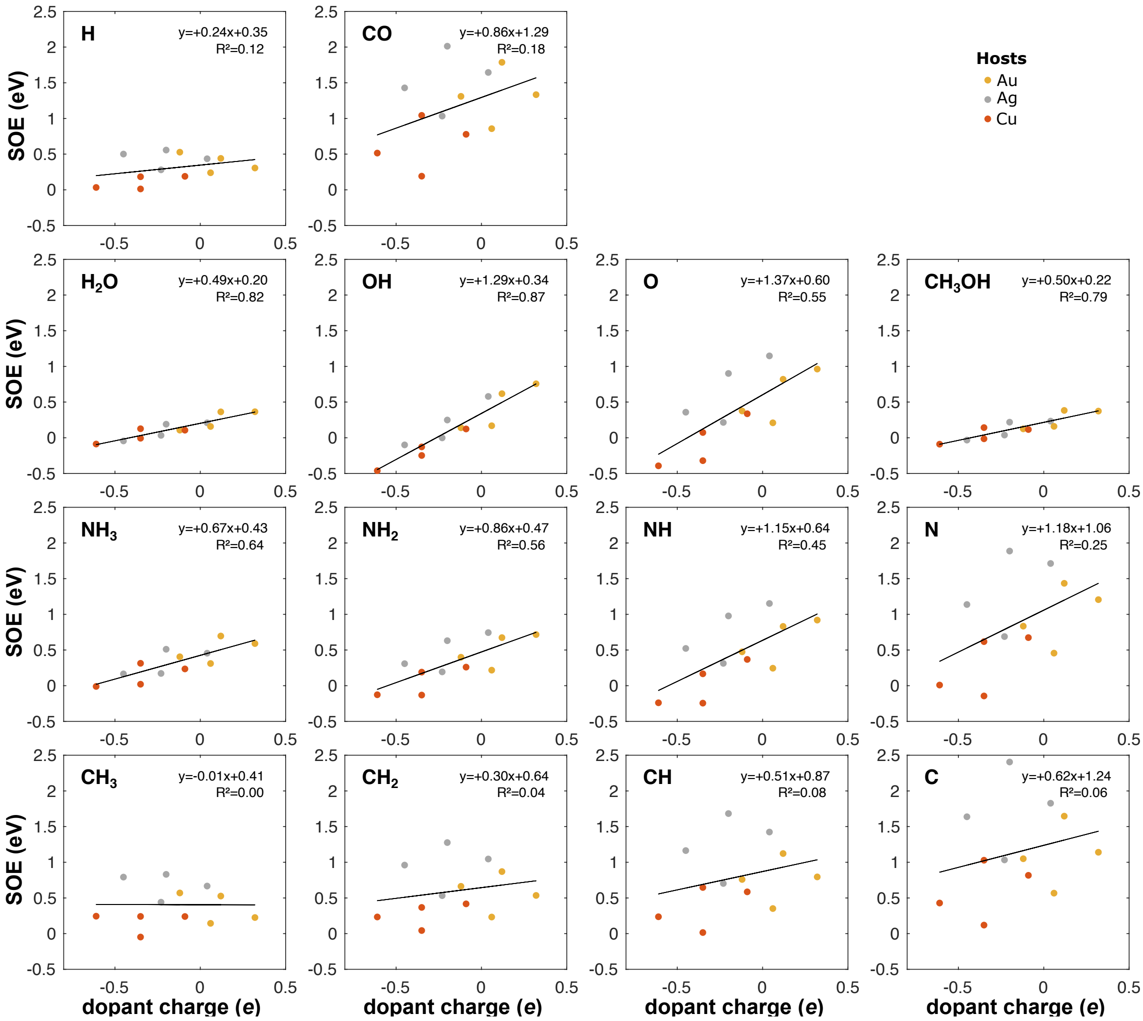

Figure S2. *Regression of the SOEs against the dopant charge (Bader approach). The line provides the least-squares linear fit. This is the same data as plotted in Figure 2, focusing on the host material rather than the nature of the adsorbates.*

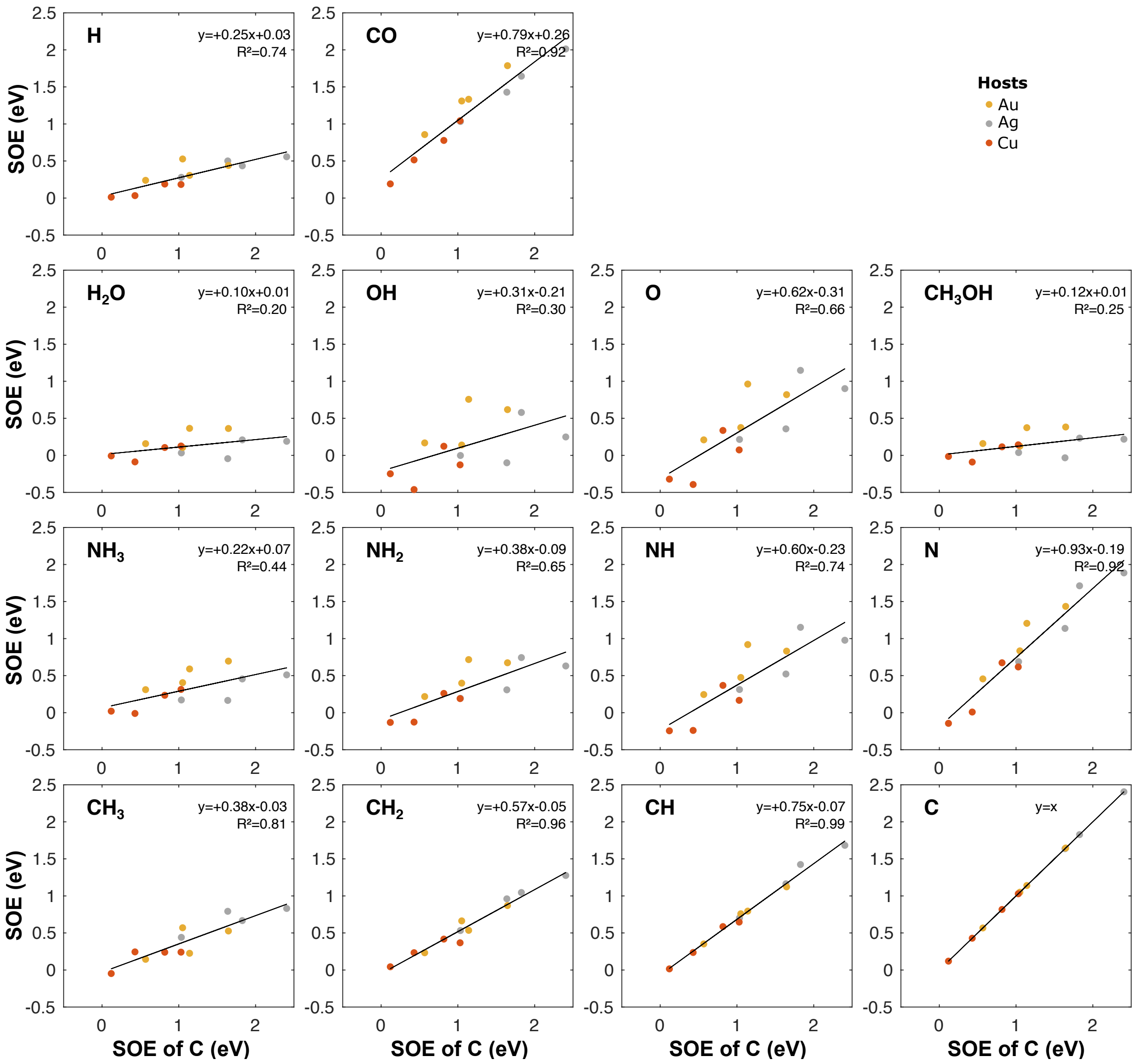

Figure S5. *Regression of the SOEs against the SOE of C. The black line provides the least-squares linear fit.*

Regarding the second point, Figures 2 and S2-S4 are only given to show the degree of correlation between the SOE and the Bader charge and the SOE of C. In Figure 2 for example, all the points would be classified as outliers for C, as the bonding mechanism is essentially covalent. But they are not actual outliers as the model is incomplete when covalent and electrostatic effects are not considered concurrently.

We have changed the caption to make this point clearer.

Figure 2. *~~Analysis of~~ Correlation between the spillover energy (SOE) and the dopant charge for different adsorbates on 12 different SAAs. (a) The SOE is defined as the energy difference between the adsorbate (in this case methyl) bonding at the dopant site (cyan) and at a distant site on the host (yellow). (b-e) SOE plotted against the dopant charge (in units of the elementary charge e) for (b) H and CO as well as other absorbates bound via their (c) O, (d) N, and (e) C atom. For each adsorbate, the least-squares linear fit is plotted as a red line.*

14. The equation in Figure S4 for C should be written as y = x

We have made the required correction (the corrected figure is provided in our answer to the previous comment).

15. What would be the difference in terms of the MAE of performing a multilinear regression with both descriptors instead of using the two-step regression approach proposed.

We have now re-analysed the data performing a more natural multilinear regression. Both the MAE and STD are improved (see new Table S5).

Additional Questions:
Urgency: High

Significance: High

Novelty: High

Scholarly Presentation: Top 10%

Is the paper likely to interest a substantial number of physical chemists, not just specialists working in the authors' area of research?: Yes
